# Longitudinal multimodal assessment of neurodegeneration and vascular remodeling correlated with signal degradation in chronic cortical silicon microelectrodes

**DOI:** 10.1117/1.NPh.7.1.015004

**Published:** 2020-01-30

**Authors:** Krystyna Solarana, Meijun Ye, Yu-Rong Gao, Harmain Rafi, Daniel X. Hammer

**Affiliations:** Food and Drug Administration, Center for Radiological Devices, Office of Science and Engineering Laboratories, Division of Biomedical Physics, Silver Spring, Maryland, United States

**Keywords:** two-photon microscopy, optical coherence tomography, angiography, implanted cortical microelectrodes, neuroinflammation, cranial window, chronic imaging window

## Abstract

**Significance**: Cortically implanted microelectrode arrays provide a direct interface with neuronal populations and are used to restore movement capabilities and provide sensory feedback to patients with paralysis or amputation. Penetrating electrodes experience high rates of signal degradation within the first year that limit effectiveness and lead to eventual device failure.

**Aim**: To assess vascular and neuronal changes over time in mice with implanted electrodes and examine the contribution of the brain tissue response to electrode performance.

**Approach**: We used a multimodal approach combining *in vivo* electrophysiology and subcellular-level optical imaging.

**Results**: At acute timescales, we observed structural damage from the mechanical trauma of electrode insertion, evidenced by severed dendrites in the electrode path and local hypofluorescence. Superficial vessel growth and remodeling occurred within the first few weeks in both electrode-implanted and window-only animals, but the deeper capillary growth evident in window-only animals was suppressed in electrode-implanted animals. After longer implantation periods, there was evidence of degeneration of transected dendrites superficial to the electrode path and localized neuronal cell body loss, along with deep vascular velocity changes near the electrode. Total spike rate (SR) across all animals reached a peak between 3 and 9 months postimplantation, then decreased. The local field potential signal remained relatively constant for up to 6 months, particularly in the high-gamma band, indicating long-term electrode viability and neuronal functioning at further distances from the electrode, but it showed a reduction in some animals at later time points. Most importantly, we found that progressive high-gamma and SR reductions both correlate positively with localized cell loss and decreasing capillary density within 100  μm of the electrode.

**Conclusions**: This multifaceted approach provided a more comprehensive picture of the ongoing biological response at the brain–electrode interface than can be achieved with postmortem histology alone and established a real-time relationship between electrophysiology and tissue damage.

## 1 Introduction

Implantable medical devices that record brain electrical activity and can deliver therapeutic electrical pulses to modulate neural signals are rapidly advancing technologies that can be used to treat a variety of neurological and psychiatric disorders, including motor and sensory deficits.^1^^–^^6^ Traditional pharmacological and biologic therapies have had limited success in treating these disorders due to the difficulty in targeting specific neural circuits with systemic strategies, as well as challenges crossing the blood-brain barrier (BBB). In contrast, brain–computer interfaces (BCIs) target local circuitry and can restore reliable communication between the brain and the external world after their connection has been compromised, either via a loss of limb, paralysis, stroke, or other motor, sensory, or cognitive impairment.^4^^–^^8^ To accomplish this significant task, BCIs must read and preprocess cortical electrical activity, extract salient features and patterns, and then translate these signals into an external command.^9^ In doing so, a BCI can circumvent the deficiency and restore functional capacities to patients, for example, to control a computer cursor to communicate or control a robotic arm and interact with the environment.^4^^,^^7^^,^^8^^,^^10^

Implanted intracortical microelectrode arrays produce higher neural signal fidelity and spatial resolution than surface electrodes shortly after implantation, but they inexorably experience more pronounced signal degradation over time that eventually leads to device failure and limits their long-term efficacy in advanced neuroprosthetic applications.^3^^,^^11^ A recent macaque study reported that over half of implanted arrays failed within the first year of implantation,^12^ and multiple others have reported a progressive decline in spike amplitudes, functional channels, and detectable single units.^10^^,^^13^ Biological responses, including BBB disruption, astrocytic and microglial encapsulation, and neurodegeneration, as well as mechanical and material failures, can contribute to decreases in the quality of the recorded signal and result in unpredictable long-term performance.^12^^,^^14^^–^^21^ Thus despite the potential of BCIs to transform neurological medicine, this uncertain long-term reliability for an implanted device is unacceptable for most clinicians and patients, even given the temporary higher benefit-risk profile for this patient population. To achieve the full potential and wide adoption of BCIs as neuroprosthetic devices will require an understanding of the causes and mechanisms of device failure as well as strategies to mitigate those failure modes.

The objective of this study was to characterize progressive neural tissue changes in response to chronically implanted cortical electrodes and investigate whether recorded electrical signals correlate with tissue damage. To accomplish this, we used a multimodal approach combining *in vivo* electrophysiology and optical imaging. Optical coherence tomography angiography (OCT-A) provides label-free visualization of surface vessels and deep capillaries as well as capillary flow,^22^^,^^23^ while two-photon microscopy (TPM) of Thy1-YFP transgenic mice allows imaging of individual layer V (L5) cell bodies and dendritic structures in fine detail.^16^^,^^24^^–^^26^ In our custom-built imaging system, the OCT and TPM beams are co-linear, affording us the unique opportunity to precisely and rapidly target the electrode location and identify and track the same neurons and vessels at multiple successive time points for over a year. This integrated approach permitted a comprehensive and longitudinal evaluation of the ongoing and dynamic biological response at the tissue–electrode interface while simultaneously tracking the fidelity of recording signals and local neural signal strength. With stable cranial window preparations for over a year, we found evidence of localized neuronal degeneration and cell loss, decreased capillary density, and reduced flow velocity near the electrode over time. Perhaps most importantly, we found that these indicators of tissue damage correlated positively with changes in electrode signal quality, thus increasing our understanding of the underlying mechanisms of BCI device signal loss.

## 2 Materials and Methods

### 2.1 Animals and Surgery

All procedures were approved by the FDA White Oak Institutional Animal Care and Use Committee and comply with the National Institutes of Health Guide for the Care and Use of Laboratory Animals. Adult male Thy1-YFP transgenic mice (B6.Cg-Tg(Thy1-YFP)HJrs/J, Jackson Laboratory, Bar Harbor, Maine, Stock No: 003782) were used in the study. Mice were 2 to 4 months old at the time of window surgery and electrode implantation or window surgery only.

During surgery, mice were anesthetized with a 4% induction dose of isoflurane (Henry Schein, Melville, New York), and then positioned in a stereotaxic apparatus (David Kopf Instruments, Tujunga, California). Mice were maintained under anesthesia with 1.5% isoflurane (0.8  L/min $O_{2}$), body temperature was maintained at ∼37°C with a thermostat-controlled heating plate (Model TC-1000, CWE Inc., Ardmore, Pennsylvania), and respiration rate was monitored and maintained at ∼100  breaths/min during the procedure. A craniotomy (∼2×3  mm) was drilled over the left motor and somatosensory cortex (coordinates relative to Bregma in mm: AP −0.5 to −3.5, L 0.5 to 2.5) using a high-speed dental drill (Osada, 0.25-mm drill bit, Osada, Inc Los Angeles, California). A custom-cut 2×2  mm glass coverslip (sterilized #0) was placed on the surface of the cortex above the dura and attached to the skull with Kwik-Sil (WPI, Sarasota, Florida) and dental cement (Parkell C&B Metabond, Edgewood, New York) on three sides.

**Fig. 1:**
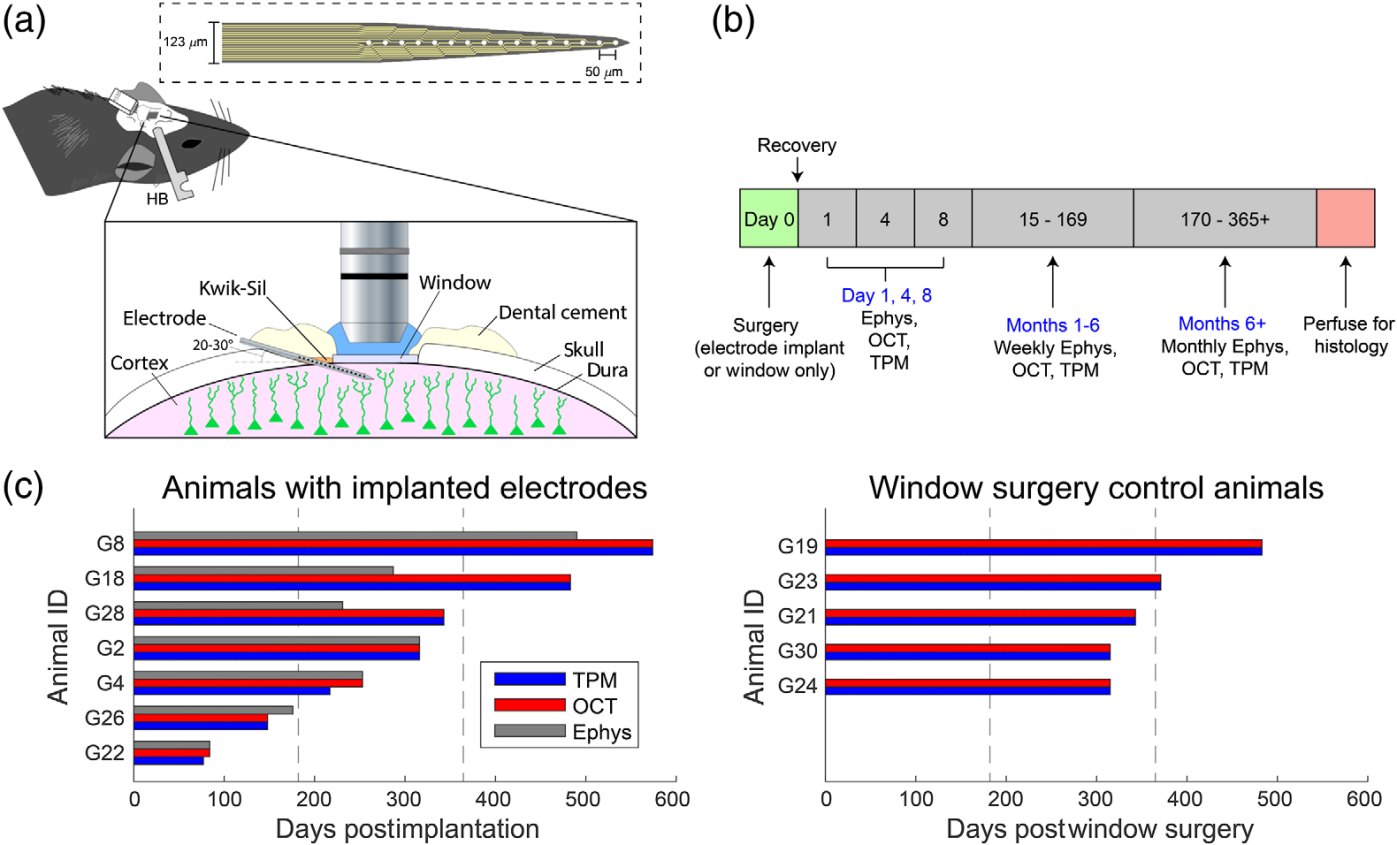
Animal preparation and imaging timeline. (a) Cross-sectional schematic of window preparation above angled (20 deg to 30 deg relative to the brain surface) electrode insertion, which allows long-term visualization of vessels and neurons. The entire preparation is sealed permanently with dental cement. HB: head-bar. Inset shows 16-channel electrode diagram. (b) Timeline of chronic imaging and electrophysiological recording following window surgery and electrode implantation on day 0. For the first week, animals were imaged on day 1 (allowing a 24-h recovery following surgery) and day 4. Beginning with day 8, animals were imaged weekly for 6 months, then monthly from month 6 to the end of *in vivo* experiments. (c) Imaging and recording duration for each electrode-implanted and window-only control animal reported in this study. Of the 12 animals used for longitudinal imaging and electrophysiology (Ephys) recording, 10 were imaged for over 6 months ($n_{\text{electrode}}=5$, $n_{\text{window-only}}=5$) and four were imaged for over 1 year ($n_{\text{electrode}}=2$, $n_{\text{window-only}}=2$).

For the electrode implantation group, a single-shank, 16-channel, Michigan-style microelectrode array (A1X16-3mm-50-177-CM16LP, Neuronexus, Ann Arbor, Michigan) was inserted into the cortex. The electrode has 16 iridium recording sites sized $177\;{\mu m}^{2}$ that were linearly distributed along the shank (15-μm thick and 123-μm maximum width) with 50-μm spacing between sites. The electrodes were inserted with recording sites facing up at an angle of 20 deg to 30 deg relative to the brain surface through the posterior edge of the craniotomy without Kwik-Sil [Fig. 1(a)]. The electrodes were inserted with a manual micromanipulator (Narishige, Amityville, New York) through the dendritic tuft and apical dendrites to the approximate depth of layer V pyramidal neuron soma (∼200 to 400  μm). A custom-made ground pin was anchored through a burr hole drilled posterior to the lambdoid suture contralateral to the craniotomy as a common reference. The exposed brain at the posterior edge of the craniotomy was covered with a thin layer of Kwik-Sil (WPI) after electrode insertion, and the glass window and percutaneous electrode array connectors were adhered to the skull permanently with dental cement. A metal bar with a screw notch was attached over the skull on the right hemisphere to stabilize the head during *in vivo* imaging. This preparation left an unobscured imaging area of ∼1.5×1.5  mm.

### 2.2 Electrophysiology Data Acquisition

To determine the electrode performance and neuronal activities over time, intracortical neural signals were acquired from freely moving mice in their home cages with a NeuraLynx Digital Data Acquisition System (NeuraLynx, Bozeman, Montana), including a DL 4SX-M 32ch Base, a headstage preamplifier HS-18-CNR-MDR50, a HS-18-N2T-16 adaptor, and Cheetah data acquisition software, at a sampling frequency of 32 kHz. High-frequency (HF) data containing multiple unit activity (MUA, filtered between 900 and 5 kHz) and local field potentials (LFP, filtered between 0.1 and 300 Hz) were saved with Cheetah software (NeuraLynx, Bozeman, Montana). Each recording session lasted about 15 min.

### 2.3 OCT and TPM Imaging

**Fig. 2:**
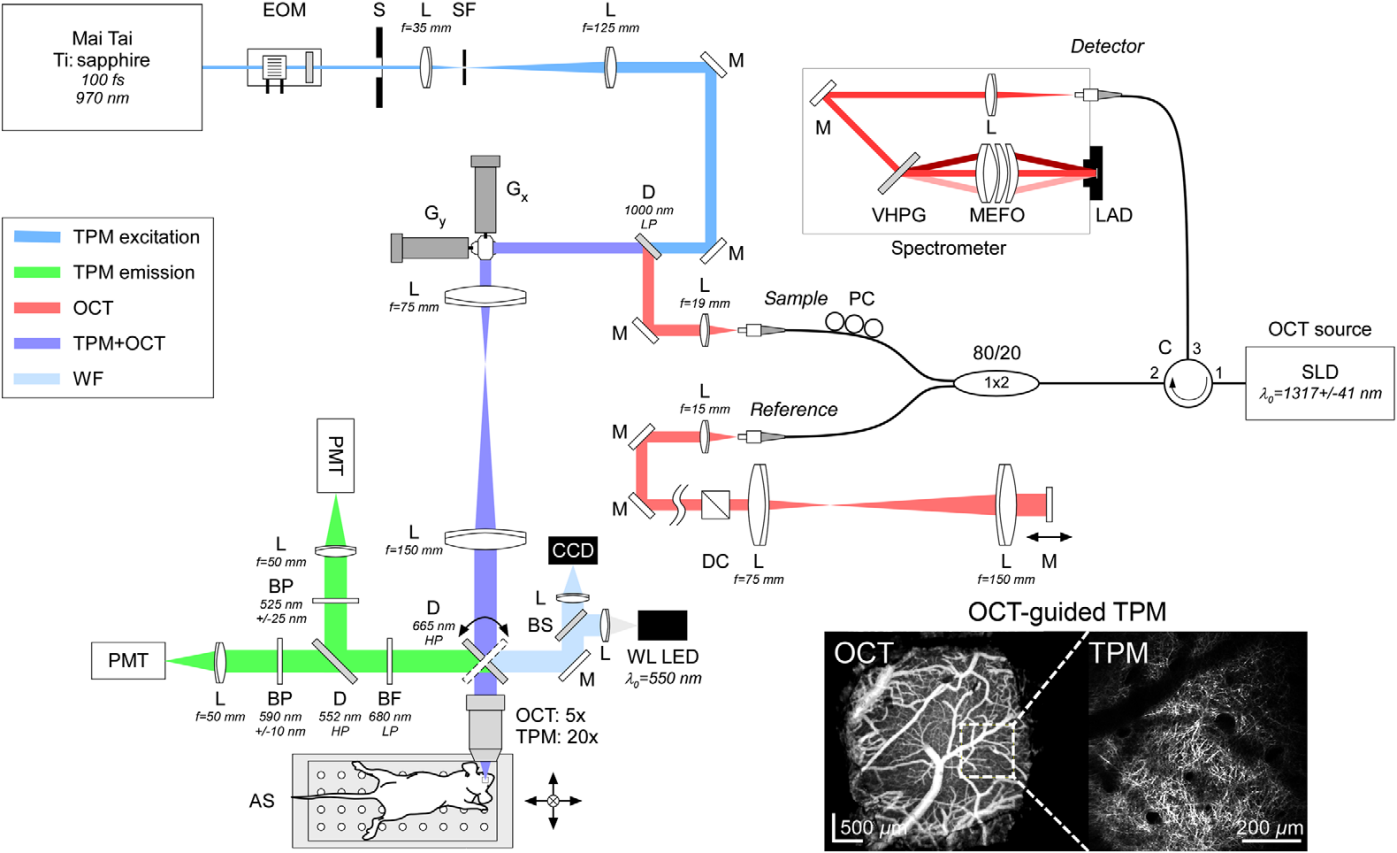
Optical schematic of a custom-built imager for OCT-guided TPM. The TPM (970 nm) and OCT (1317 nm) beams are co-linear. Animal is positioned on a computer-controlled, motorized three-axis AS. EOM, electro-optic modulator; S, shutter; SF, spatial filter; L, lens; M, mirror; D, dichroic beamsplitter; Gx and Gy, galvanometers; BS, glass beamsplitter; BP, band-pass filter; PMT, photomultiplier tube; WL LED, white light light-emitting diode; CCD, charge coupled device camera; DC, dispersion cube; 1×2, fiber coupler; C, circulator; PC, polarization controllers; VHPG, volumetric holographic phase grating; MEFO, multielement focus objective; LAD, linear array detector; and SLD, superluminescent diode.

Animals were imaged on a custom-built imager with TPM and OCT channels in which the TPM (970 nm) and OCT (1317 nm) beams are co-linear. Figure 2 shows the optical schematic for the combined system. For the imaging protocol, mice were anesthetized with a 4% induction dose of isoflurane and then maintained with 1% to 2% of isoflurane throughout imaging. Body temperature was maintained at 37°C on a heating pad. The animal was positioned for OCT and TPM imaging using a three-axis motorized animal stage (AS) (Thorlabs Inc., Newton, New Jersey).

The OCT channel uses a broadband superluminescent diode (Exalos, Schlieren, Switzerland) for illumination with a center wavelength of 1317 nm and a 3-dB bandwidth of 83 nm, which produces a theoretical axial resolution of 9.2  μm in air (6.7  μm in tissue with refractive index n=1.38). The OCT channel is arranged in a standard spectral domain configuration, with a circulator and 1×2 coupler (AC Photonics Inc., Santa Clara, California) for efficient fiber interferometry. A spectrometer (Wasatch Photonics Inc., Durham, North Carolina) calibrated to the source with a center wavelength of 1290 nm and bandwidth of 197 nm is used for spectral interferometry. The spectrometer uses an InGaAs linear detector (UTC Aerospace Systems/Sensors Unlimited, Princeton, New Jersey) with a maximum line rate of 147 kHz (75 kHz used in this study for all scans). The OCT beam is directed into the front-end imaging optics just before the galvanometer pair with a low-pass dichroic beamsplitter that cuts at 1000 nm (Thorlabs). For OCT imaging, a 5× telecentric objective (Thorlabs) is used. The axial and lateral point spread function, measured with a nanoparticle-embedded phantom,^27^ was ∼10.2 and 6.2  μm in tissue with a depth-of-focus of ∼300  μm. The reference arm contains a cube and lens pair that matches the dispersion properties of the objective and lens pair in the front-end optics.

Reflectance, angiography, and flow videos were acquired and processed in near real time as described previously^23^ using custom software written in LabVIEW (National Instruments, Austin, Texas), MATLAB (Mathworks, Natick, Massachusetts), and C/C++ and using the CUDA parallel processing platform (NVIDIA, Santa Clara, California) on the system computer’s Tesla K40 GPU (graphical processing unit) video card (NVIDIA). The reflectance video used standard OCT processing. The angiography video was calculated from the average absolute difference of multiple B-scans collected at each location,^28^ and the flow video was calculated using the capillary velocimetry algorithm reported by Srinivasan et al.^29^ Two sets of volumetric scans were taken with OCT. The first set, taken with a lower pixel density of 4  μm/pixel, was two 2×2  mm scans (500×500 lateral pixels, excluding flyback) covering the entire window with the focus just beneath the window (∼0- to 50-μm deep) and ∼300- to 400-μm deep, the latter roughly at the depth of the electrode tip. OCT reflectance and angiography videos were collected for these sets, where 10 B-scans (interval=7.04  ms) were collected at each lateral position. The scans were used to measure light reflectance changes associated with electrode implantation, to visualize superficial vascular changes associated with window implantation, and to further guide OCT imaging and TPM alignment. The second set, taken with a higher pixel density of 2  μm/pixel, was three 0.5×1.5  mm strip scans (250×750 lateral pixels, excluding flyback) with 50  μm lateral overlap, which were combined to form a single ∼1.5×1.5  mm high-density montage of the window region centered laterally on the electrode with the focus set approximately at the electrode tip depth (∼300 to 400  μm beneath the window). For the montage scans, reflectance, angiography, and flow videos were acquired, where 100 B-scans (interval=3.76  ms) were collected at each lateral position. Capillary velocimetry was calculated with a moving window correlation width of 8 B-scans (30 ms). This set of high-pixel density images was used to analyze deep capillary structure and flow velocity.

TPM imaging of the neuronal structure was performed immediately following OCT imaging. The TPM channel uses a Mai Tai titanium:sapphire femtosecond laser source (Spectra-Physics, Santa Clara, California) tuned to a center wavelength of 970 nm with 100-fs pulse duration for excitation. An electro-optic modulator (Conoptics Inc., Danbury, Connecticut) controls illumination laser power. The beam is first expanded to ∼5-mm diameter before the scanning galvanometers and again to ∼13-mm diameter before the objective. A 20× water immersion objective (Nikon, Tokyo, Japan) with a numerical aperture (NA) of 1 was used for TPM imaging. The spot size measured with a fluorescent microsphere standard (TetraSpeck, Molecular Probes Inc., Eugene, Oregon) was ∼1  μm. The visible wavelength fluorescent emission light excited with the two-photon process was directed without descanning to the photomultiplier tubes (PMT, Hamamatsu, Japan) with a low-pass dichroic beamsplitter (670-nm cut, Semrock) and then split by an additional dichroic beamsplitter (552 nm cut, Semrock) to the two PMTs. Additional bandpass filters at 525 and 590 nm (Semrock) were placed before the two PMTs. Custom code (LabVIEW) controlled the hardware and image acquisition. Image stacks were collected at 2 fps from the brain surface to a final depth at which signals could no longer be detected (typically 500 to 700  μm), with a 2-μm z-step size. Laser power was automatically increased linearly (or logarithmically) during depth stepping to account for exponential tissue light penetration losses. The field of view (FOV) was set to 476×476  μm for all TPM stacks with 500×500  pixels. In each imaging session, four stacks were taken to form a 776-×776-μm montage around the electrode region (or around a control region for the window-only animals) with 176-μm overlap.

The OCT channel served to guide subsequent two-photon imaging as follows (see inset of Fig. 2 and Fig. S1 in the Supplementary Material). Because the two-photon and OCT beams are aligned co-linearly, the lateral position of the two-photon FOV on the cortex could be quickly identified after OCT imaging. After the OCT 1×3 montage was collected, the animal stage was set to the middle of the window and the objectives were switched to change from wide-field OCT imaging to narrow field TPM imaging. The 2×2  mm OCT scan with focus set just below the window was processed, revealing both the surface vasculature map and the position of the electrode, and imported into the TPM software. An overlay on the OCT *en face* image enabled rapid location of the electrode, where the 2×2 TPM montage could then be immediately collected. This was particularly important because the electrode appeared dark on the TPM image and indistinguishable from vessels or other gaps between neurons, while in OCT it appeared bright because the metallic surface, especially of the electrode pads, reflected back to the OCT detector. The OCT-guided TPM arrangement allowed individual neurons, their dendritic processes, and capillary segments to be tracked long term, for up to 1.5 years. The alignment time for TPM imaging was also significantly reduced with OCT-guidance.

### 2.4 Electrophysiological Data Analysis

**Fig. 3:**
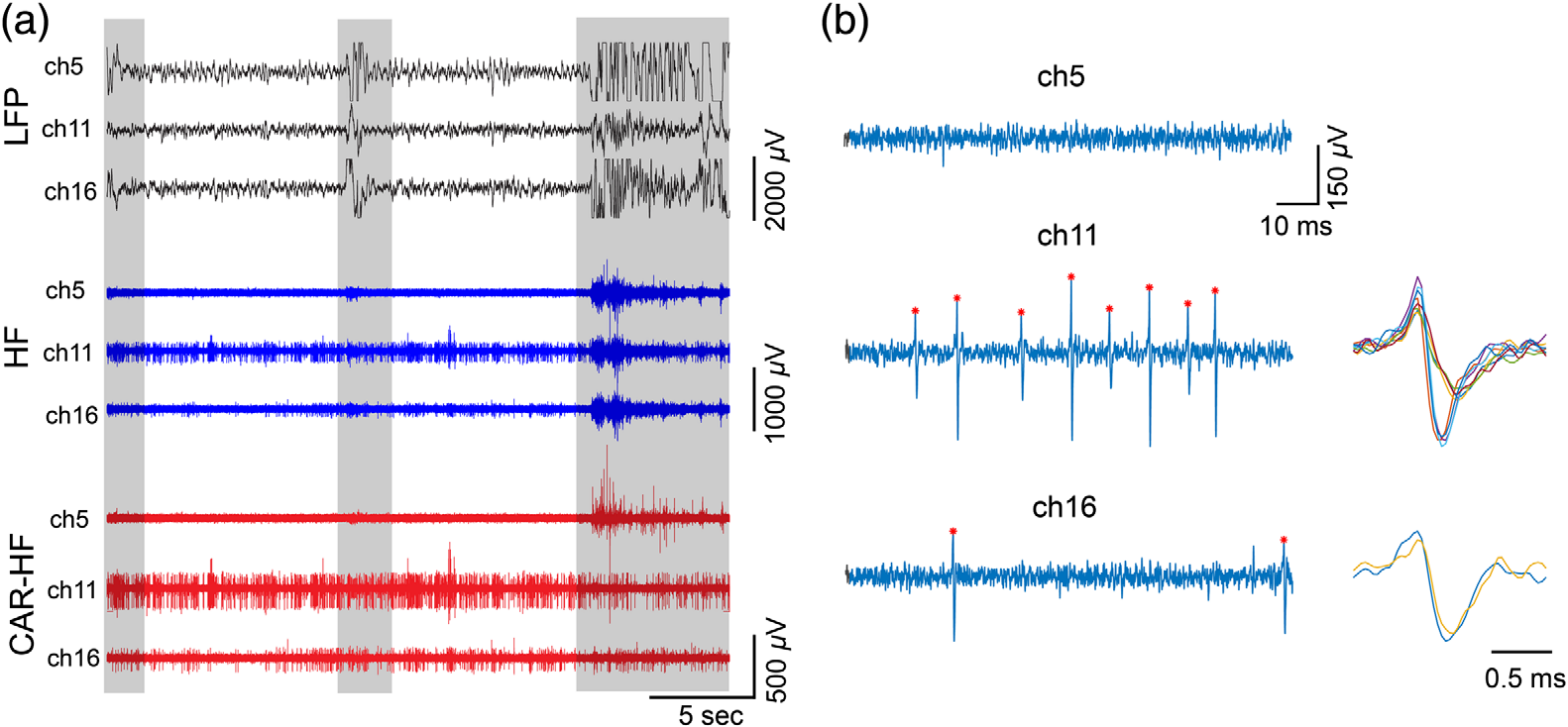
Example electrophysiological recordings. (a) Example raw LFP, HF, and CAR-HF traces from three representative channels (ch5, 11, and 16). Channel number portrays sequential electrode site order. Channel 1 represents the electrode site nearest the pial surface, whereas channel 16 is the deepest electrode site. Shaded areas show recording segments contaminated by myoelectric and movement artifacts, which were removed from further LFP and MUA quantitative analysis. (b) Example spikes detected in 100 ms recordings shown in (a). No spikes were detected in ch5 in the segment. Two types of waveforms of spikes were detected in ch11, presumably from two different neurons.

LFP and HF data were imported into MATLAB (Mathworks) for manual removal of segments with myoelectric and/or movement artifacts. Signals were scored for the presence of artifacts when unusually high-amplitude and low-frequency events occurred in LFP and when clusters of high-amplitude spindle-shaped HF oscillations occurred in HF signals across all channels [Fig. 3(a)]. Only snippets with ≥4  s artifact-free data and recording sessions with ≥5 clean snippets were used for quantitative analysis. Starting from 15 min total recording time (900 s), the total duration of clean recordings was 162±130  s (range: 26 to 695 s). When saturated LFP and extraordinarily high noise in HF signals were present in a particular channel throughout the recording, that channel was considered not viable. In our previous investigation with simultaneous brain electrophysiological recording and movement tracking, we observed a close correlation between an animal’s movement status and the presence of artifacts.^30^ Therefore, the data used for MUA and LFP analysis in this study were primarily from the quiescent state.

For MUA analysis, we estimated the standard deviation (STD) based on the mean of bandpass-filtered, artifact-free, and common-average-referenced (CAR) HF signals. Then we used five STDs as an amplitude threshold for spike detection [Fig. 3(b)]. Spike rate (SR) was calculated by dividing the total number of spikes by the length of the artifact-free recording. Total SR was the sum of SR across all 16 channels. Signal-to-noise ratio (SNR) was calculated by dividing the average spike amplitude by one STD of the signal mean. Spike amplitude was defined as the positive peak voltage from baseline. For LFP analysis, data were downsampled to 2 kHz. Power spectral density (PSD) for each recording session was computed with a multitaper fast Fourier transform (NW=3, k=5) estimate using MATLAB functions from the Chronux Toolkit.^31^ Absolute power was obtained for the δ(1 to 4 Hz), θ(4 to 8 Hz), α(8 to 13 Hz), β(13 to 30 Hz), low-γ (30 to 56 Hz), and high-γ (64 to 100 Hz) frequency bands. To exclude the possible effect of 60 Hz noise on the power estimation, signals between 56 and 64 Hz were removed from further quantification (rather than applying a notch filter).

### 2.5 OCT Image Analysis

Each acquired OCT set yielded two or three videos (volumetric scans)—reflectance, angiography, and flow—depending on whether flow data were processed (Fig. S1 in the Supplementary Material). Each video frame is an average B-scan (x−z plane) at a single lateral (y) position. The videos were further processed, and montages were aligned using custom software written in LabVIEW. Axial projections [average intensity projection (AIP) or maximum intensity projection (MIP)], defined as the average or maximum pixel value in a single axial line projected through a three-dimensional (3-D) volume, were calculated to produce *en face* (x−y plane) images of the superficial vasculature or deeper capillaries. For the lower density scans, 200-μm-thick AIP angiography maps of the superficial vasculature were produced. For the higher density montage, 100-μm-thick MIP angiography and flow velocity maps above and below the electrode and a 200-μm-thick MIP angiography and flow velocity map of the entire depth region (i.e., electrode centered axially) were collected. Generally, further processing was only performed on the 200-μm-thick *en face* images. The images from different days were then collected into time-lapsed videos and registered using either ImageJ’s (NIH, Bethesda, Maryland) StackReg plugin^32^ or custom software, using a rigid body transformation (translation and rotation). See Videos S1 and S2 [URL: https://doi.org/10.1117/1.NPh.7.1.015004.1 and https://doi.org/10.1117/1.NPh.7.1.015004.2] for an example of superficial vessels and deep capillary from an example animal measured with OCT angiography.

In general, for both OCT and TPM analysis, normalization of absolute measurements is necessary to account not only for the wide natural interanimal anatomical and physiological variability but also for differences in analysis, for example, the size of regions-of-interest (ROIs) due to electrode masking. For our imaging protocol, normalization is best calculated with respect to the first imaging day (1 day postimplantation, dpi), where the changes were closest to baseline. Except where noted, the measured parameter (x) was normalized to the first day ($x_{0}$) using $(x-x_{0})/x_{0}$ (zero baseline).

To characterize vessel intraluminal diameter, vessel density, and capillary flow velocity, the time-lapsed videos were further processed using custom software. The ROIs in the following description were chosen to avoid very large superficial vessels and any regions where dura/bone regrowth occurred under the window over time. Also the ROIs on the electrode animals were specifically designed to mask the electrode region (which could be quite large due to electrode movement over time, see Fig. S4 in the Supplementary Material). The vessel density of the superficial vasculature was measured with square ROIs sized 0.4×0.4  mm (100×100  pixels) and 1×1  mm (250×250  pixels). The capillary density and flow velocity for the deeper montages were measured with a square ROI sized 0.8×0.8  mm (400×400  pixels) and three annular regions centered approximately on the electrode tip measuring 0 to 100  μm, 100 to 200  μm, and 200 to 300  μm.

The vessel diameter was measured on 5 to 7 capillary segments from the deep capillary montage time-lapsed videos for each animal. Vessels that had clear wall boundaries on the first day (for normalization) and over many days in the time-lapsed period were selected. The full-width half-maximum diameter measurements were calculated from an average profile calculated over five adjacent lines in an ROI manually positioned perpendicular to the vessel direction. Measurements were discarded when the vessels moved outside of the ROI (from registration errors or local movements) or out of the depth-of-focus or when the measurement algorithm could not distinguish the vessel segment of interest from adjacent segments. The algorithm to calculate ROI vessel density from the MIP angiography maps used two measures. First, the region was thresholded using a threshold value that was an empirically chosen ratio (0.9) of the mean pixel intensity of the entire region. The subsequent binary image was processed with particle filtering (0 to 15 pixel regions rejected) and summed. This analysis (“vessel pixels”) reveals capillary density, that is, each count of the total represents a pixel where a capillary exists, irrespective of the flow velocity in the capillary. However, because the analysis relies on thresholding, inaccuracies can occur for poorly chosen threshold values. The second type of analysis (“ROI pixel sum”) relies on the understanding that any signal in the OCT angiography maps derives from motion, and movement artifacts as distinct from blood flow are minimal in these datasets. This analysis simply sums the pixel gray-scale intensities in the ROI. However, this is not necessarily a true measure of capillary density, but rather a measure of the convolution of flow and capillary density, because high-pixel gray-scale values correspond to higher flow, although in a nonlinear fashion.

The capillary flow processing algorithm calculates flow velocities by first converting the gray-scale flow maps from Δf to velocity (mm/s) using the linear calibration parameters found in a separate system calibration measurement. That measurement used a uniform scattering phantom (Spectralon, Labsphere Inc., North Sutton, New Hampshire) and a precision linear stage moving at a constant velocity. From the flow data, four measures were extracted: average flow velocity, which is the mean value of all flow velocity pixels within the ROI (excluding pixels outside of the linear range); flow velocity mode, which is the flow velocity value with the maximum count in the histogram; total flow count, which is the count of all flow velocity pixels in the ROI; and total flow velocity, which is the sum of the flow velocity values for all ROI pixels (and equivalently is equal to the average flow velocity×the total flow count).

### 2.6 TPM Image Analysis

The four 476-×476-μm image stacks were combined to form a full 776-×776-μm montaged FOV encompassing the electrode region (or an equivalent control region for window-only animals) using custom code (LabVIEW). From this 3-D stack, an MIP image was then generated from the 50 slices (100  μm) where layer V somas were visible. MIP images from each imaging day per animal were concatenated and registered over time using ImageJ software (StackReg, rigid body) to monitor changes in cell numbers around the electrode. Cells were manually identified and selected using ImageJ’s “Point Tool” in each full FOV image and automatically added to the ROI manager. The coordinates of each cell were then used to calculate the distance from the electrode tip and used to determine cell number changes within the full field and at specific distances (0 to 100  μm, 100 to 200  μm, 200 to 300  μm, and 300 to 400  μm).

The TPM and OCT analyses using annular ROIs on two-dimensional (2-D) axial projections (i.e., MIPs) were designed as a simplified test of the hypothesis that biological changes take place as a function of distance from the electrode. This hypothesis is most accurately achieved by consideration of the 3-D cylindrical angled insertion region around the shank. We justify our simplification for the TPM volume by noting that the neuronal somas are confined to a limited depth range (∼100  μm), which is readily captured in the axial projection. For the OCT volumes, the depth-of-focus (∼300  μm) limits the analyzable region, which again is readily captured in angiography and flow maps via MIP. (OCT volumes were acquired without focus tracking inherent to TPM acquisition due to practical limitations in terms of the dataset size and acquisition time.) Therefore, because of the limited OCT depth-of-focus and the anatomical confinement of neuron somas, we chose to analyze lateral annular regions on 2-D projected regions rather than the 3-D cylindrical space around the inserted shank.

### 2.7 Histology

At the culmination of the imaging protocol, mice were deeply anesthetized with sodium pentobarbital (100  mg/kg, i.p.) and perfused transcardially with saline and then 10% formalin in phosphate buffered saline (PBS) (Fisher Scientific, Hanover Park, Illinois). Brains were extracted and cut to 50-μm-thick coronal slices using a vibrating microtome (Campden Instruments, Lafayette, Indiana). Free-floating sections were washed in 1× PBS and incubated in a 0.3% Triton X-100 and 4% normal goat serum (NGS, Millipore Sigma, St. Louis, Missouri) solution for 1 h at room temperature to permeabilize the membrane and block nonspecific binding. Slices were then incubated overnight on a shaker at room temperature with primary antibodies against NeuN (pChicken; Millipore Sigma) to stain neuronal cytoplasms, GFAP (mRat; ThermoFisher, Waltham, Massachusetts) for astrocytes, and Iba1 (pRabbit; Wako, Osaka, Japan) for microglia, each at 1:500 dilutions in 0.3% Triton X-100/ 4% NGS in 1× PBS solution. The following day, slices were washed in PBS, then incubated for 1 h at room temperature with corresponding secondary antibodies raised in goat: Alexa Fluor 405 (antichicken; Abcam, Cambridge, Massachusetts), Alexa Fluor 594 (antirat, ThermoFisher), and Alexa Fluor 647 (antirabbit, ThermoFisher), each at 1:1000 dilutions in PBS. Stained slices were mounted on gelatin subbed microscope slides and cover-slipped with Fluoromount (Southern Biotech, Birmingham, Alabama). Fluorescent images were obtained with an Olympus (Center Valley, Pennsylvania) FV1000 confocal microscope.

## 3 Results

Craniotomies were performed on 24 Thy1-YFP transgenic mice. Animals were divided into two cohorts, those implanted with a window only (control group) and those implanted with a window and single-shank Michigan-style depth electrode [electrode group, Fig. 1(a)]. Among the total, 12 animals (8 electrode, 4 window-only) were terminated acutely (within 1 month) due to surgical complications and poor cranial window visibility, either from gaps between pial surface and window or small superficial bleeds. No images from the acutely sacrificed animals were analyzed though electrophysiology signals were assessed in this acute group in an attempt to predict tissue responses that can prevent long term use. Among the 12 animals imaged and recorded over a long term (>3 months), the window-only group contained 5 animals and the electrode group contained 7 animals. After surgery, longitudinal OCT/TPM imaging and electrophysiological recordings were performed periodically [as indicated in Fig. 1(b)]. To investigate the contribution of the neuronal structure and vasculature changes following electrode implantation to electrode performance, OCT and TPM images were collected on the same day as intracortical electrophysiological recordings. Imaging was always performed after electrophysiological recording to minimize the effect of isoflurane on brain signals.

The length of time each animal remained in the experiment was variable and is shown in Figs. 1(c) and 1(d). A common observation across all animals was dura thickening and periosteum or skull regrowth under the cranial window. As this occurred in both window-only and electrode animals and has been reported previously,^22^^,^^33^^,^^34^ it is considered an artifact of the window implantation surgery. Regrowth generally began at the edges of the window and gradually spread over time. Several animals were terminated before the 1-year endpoint if the superficial regrowth occluded deeper visibility in the specific regions of interest imaged over time. One electrode-implanted animal (G22) was terminated after 3 months because the steep angle of the electrode was mechanically blocking the two-photon objective from acquiring deeper images, although OCT imaging and electrophysiology recordings were unaffected.

### 3.1 Chronic Electrode Signal Degradation

To determine the viability of electrodes and neuronal activity, we performed intracortical signal recording from freely moving electrode-implanted animals. We analyzed longitudinal LFP and MUA data from six animals where OCT and TPM images were acquired for durations greater than ∼6 months. Suspicious mechanical/material deterioration of electrodes, manifest as saturated LFP and extraordinarily high noise in HF signals, started to appear in some channels in each array between 245 and 323 dpi. Entire arrays stopped recording analyzable neuronal signals between 315 and 462 dpi.

**Fig. 4:**
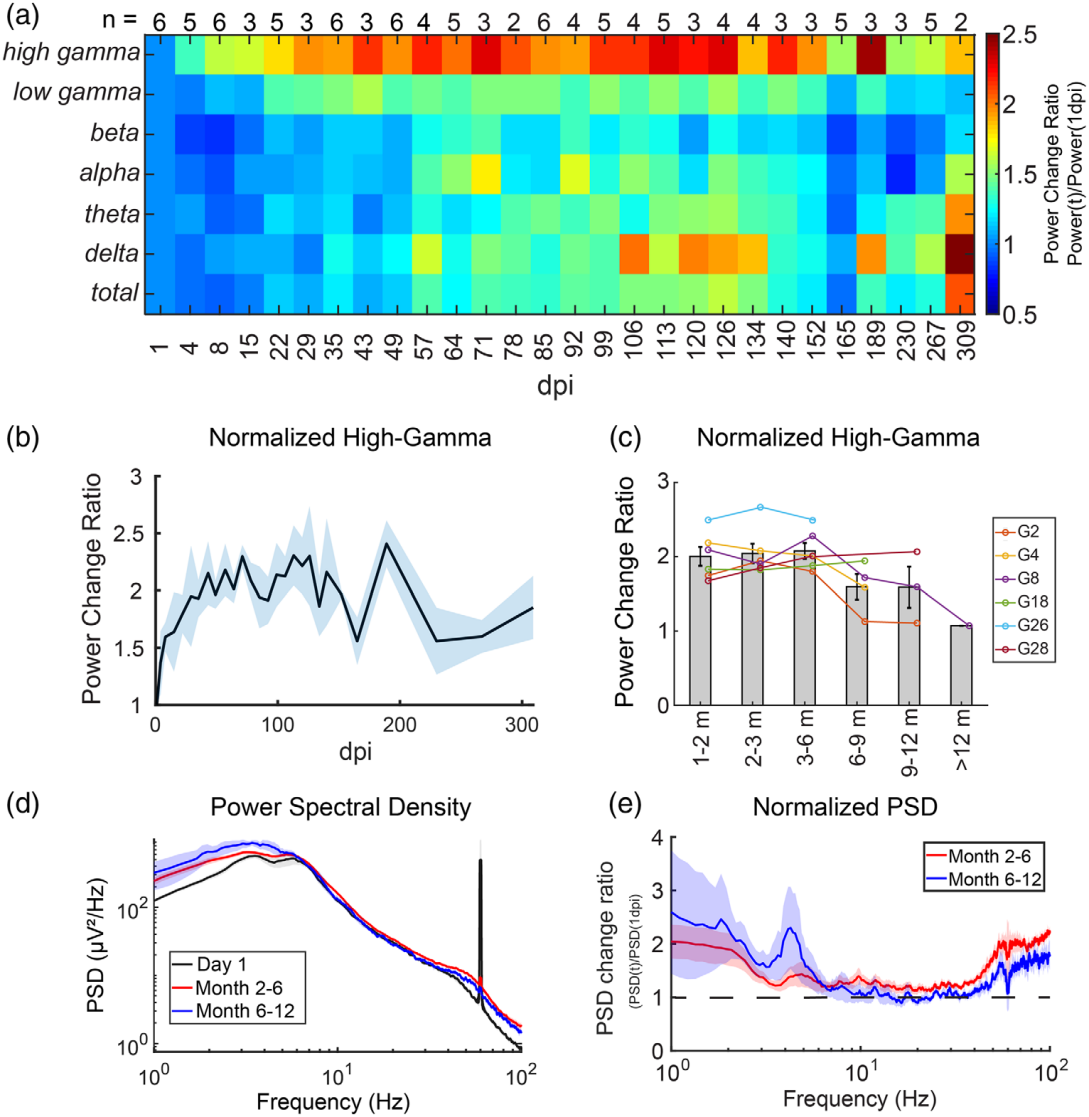
Chronic changes in LFP spectral components. (a) Average changes in power at different frequency bands from 1 dpi, calculated by dividing the power at postimplantation dates by that of 1 dpi. Numbers on the top row show the number of animals at that specific time point. Some dates had <6 animals due to the lack of enough artifact-free signals (see Sec. 2 for criteria). Note the increase in high-gamma signal throughout recording duration and the increase in delta oscillations at chronic time points. (b), (c) The trend of change in high-gamma power normalized to 1 dpi with SEM indicated by shadowed area in (b) and error bars in (c). (d) Average PSD at 1 dpi, 2 to 6 months, and 6 to 12 months postimplantation. (e) PSD normalized to 1 dpi averaged at 2- to 6-month and 6- to 12-month windows. Note the increase at low-and high-frequency bands, but the change in other frequency bands are less prominent. Shaded area for all figures indicates ±SEM.

For LFP, animals demonstrated a consistent and gradual increase in the high-gamma frequency band two weeks after the implantation surgery until reaching a relatively steady state at the beginning of the second month, then remained stable until after 6 months of implantation [Figs. 4(a) and 4(b)]. This suggests a recovery from implantation surgery. After 6 months, some animals showed a small reduction in high-gamma activity toward day 1 postimplantation levels [Figs. 4(c)–4(e)]. This can possibly be attributed to aging^35^ or neuronal cell loss induced by the electrode implant (discussed in Sec. 3.2). In addition, slow delta oscillations gradually increased at chronic time points. This is most likely due to aging according to a recent report^35^ and our findings.^30^

**Fig. 5:**
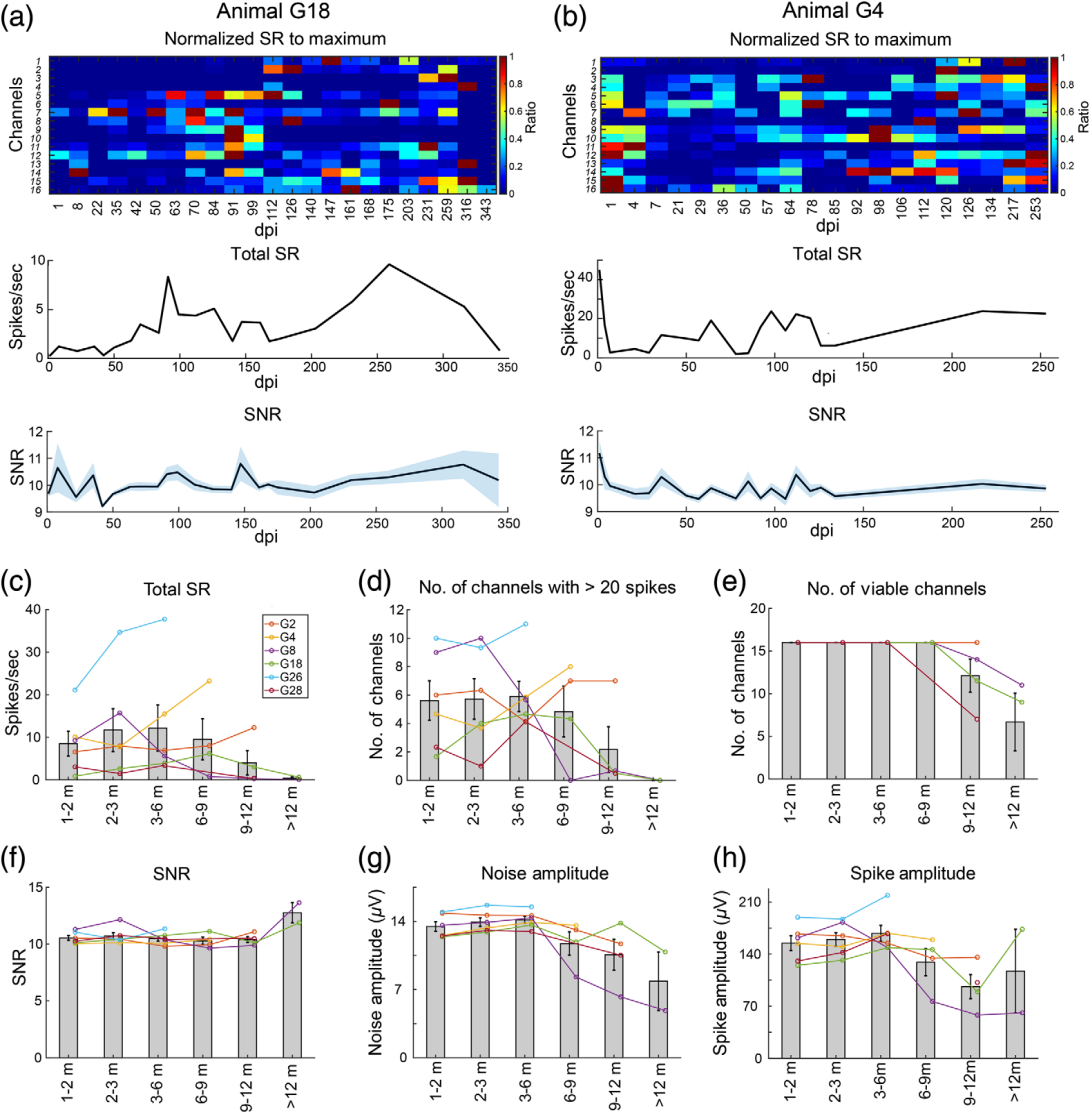
Chronic MUA changes. (a) An example animal with an acute increase in tSR. Top panel shows the SR normalized to the maximum SR recorded by each specific channel throughout the experiment. Channel number portrays sequential electrode site order, remapped from the Neurolynx channel numbers. Channel 1 represents the electrode site nearest the pial surface, whereas channel 16 is the deepest electrode site. Middle panel is the tSR recorded by all 16 channels, summed across all channels. Bottom panel shows the SNR over time. (b) A representative animal with an acute decrease in tSR. Same as (a), panels show normalized SR of 16 channels, tSR, and SNR over time. (c) Total SR over time for all six implanted animals with long-term electrophysiological data. Data are averaged within monthly or quarterly time windows for better visualization of the trend of change. tSR started reducing 6 to 9 months postimplantation. After 12 months of implantation, electrodes almost completely failed to record spikes. (d) Number of channels recording more than 20 spikes in a recording session, which shows a similar trend to tSR. (e) Number of viable channels was determined by the quality of LFP and MUA (see Sec. 2 for criteria). Electrodes started to physically fail 9 to 12 months postimplantation, which is after the reduction in SR. (f) SNR remains stable for up to 12 months postimplantation. (g) Amplitude of noise, shown as one STD of the signal mean, started reducing 6 to 9 months postimplantation. (h) Change of amplitude of spikes, which, in line with noise amplitude, started decreasing from 6 to 9 months postimplantation. Data expressed as mean±SEM.

For MUA, we compared total spike rate (tSR), the SR sum across all 16 channels, across all time points for each animal. Acute changes in tSR varied between animals. Three animals showed a reduction in tSR in the first month of implantation, whereas the other three animals had increased tSR [Figs. 5(a) and 5(b) shows examples of each]. The trend of acute tSR change appears inversely correlated with the absolute tSR on the first day postimplantation, with a higher tSR on the first day associated with an acute decrease, whereas a lower initial tSR was followed by an acute increase. After the first month, tSR started to gradually increase in all animals, reached a peak at about 3 to 9 months, and then decreased [Fig. 5(c)]. The reduction in tSR was associated with a decrease in the number of channels that recorded more than 20 spikes throughout a recording session [Fig. 5(d)]. Unlike tSR, SNR remained stable after one month postimplantation [Fig. 5(f)], which was associated with a chronic reduction in amplitude of both noise and spikes after 6 months implantation [Figs. 5(g) and 5(h)]. To understand the effect of SR on noise amplitude that was expressed as one STD of the mean in Fig. 5(g), we compared STD of the mean with that of the median. No notable difference was detected in the trend of change between STDs of the mean and median (data not shown), suggesting that the effect of SR on noise amplitude is negligible.

It is well accepted that both biological and nonbiological factors affect the longevity of electrodes. The observation that high-gamma activity and tSR begin to decline at 6 to 9 months postimplantation, preceding the electrode failure which did not occur until 9 to 12 months, suggests that biological failure might occur before mechanical/material deterioration of electrodes.

### 3.2 Neuronal Somatic and Dendritic Atrophy with Long-Term Electrode Implantation

**Fig. 6:**
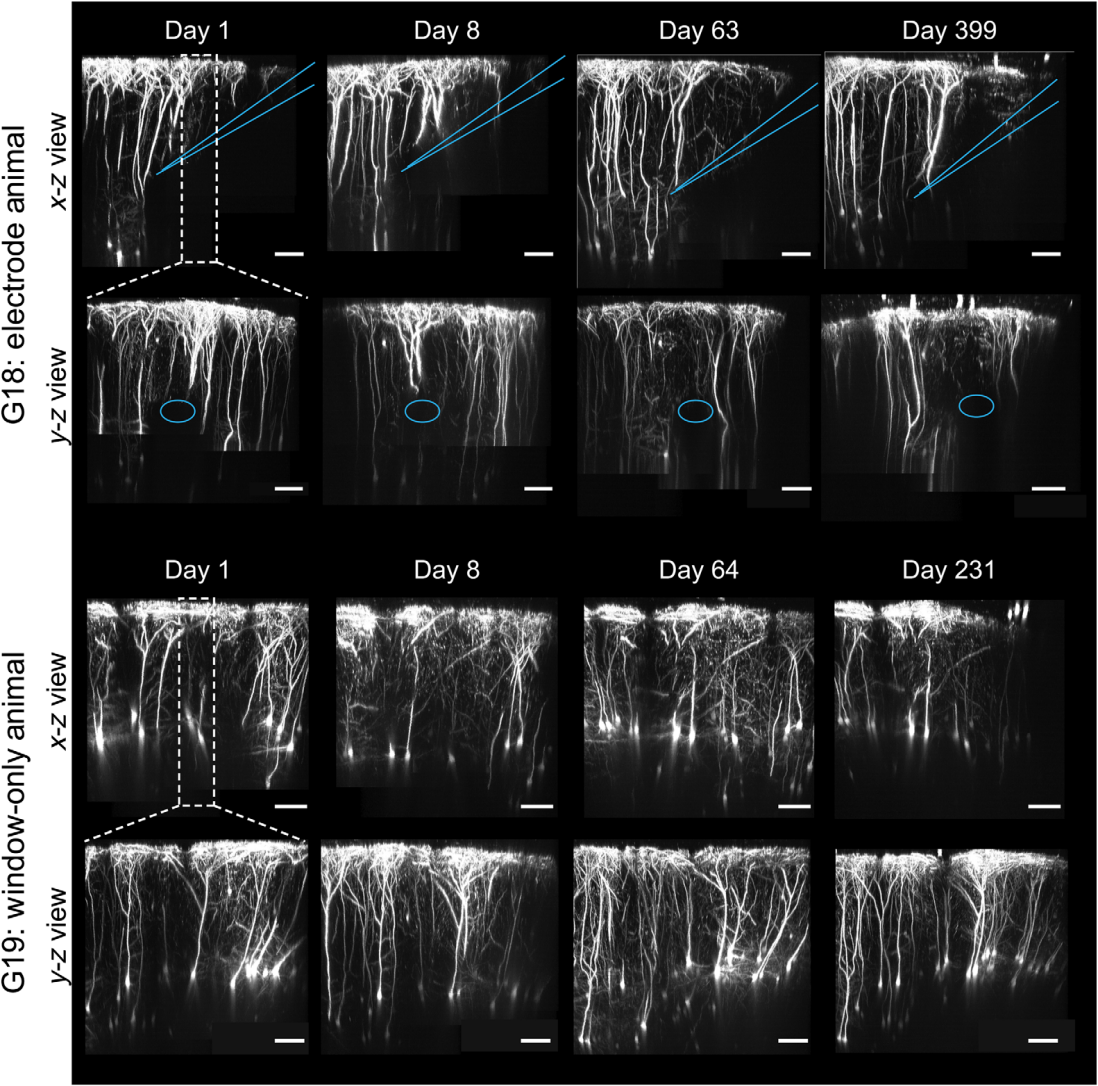
Transected dendrites in the electrode path atrophy over time. 2-D MIPs for an example electrode-implanted animal (top half) and window-only control (bottom half). For each animal, slices parallel to the electrode path (x−z view; 50  μm) and perpendicular to it (y−z view, 100  μm) are shown. For the electrode-implanted animal, blue lines show the side view of the electrode (in the x−z view), and a blue oval shows a cross section of the electrode (in the y−z view). Severed dendrites in the electrode path are evident at 1 dpi, although dendritic tuft arborization remains complex immediately after this acute injury. Clear evidence of neurite fragmentation and reduced arborization is seen as early as 63 dpi, and dendrites do not recover a year postimplantation.

To investigate the longitudinal neural response to electrode implantation, we used repeated two-photon imaging over time to acquire high-resolution 3-D stacks of fluorescently labeled cortical neurons (see Videos S3 and S4 [URL: https://doi.org/10.1117/1.NPh.7.1.015004.3 and https://doi.org/10.1117/1.NPh.7.1.015004.4]). Thy1-YFP transgenic mice express yellow fluorescent protein in layer V (L5) pyramidal neurons, axons, and their dendritic arbors.^36^ Thus we were able to visualize structural changes as early as 24 h postimplantation and for durations exceeding a year. Figure 6 shows 2-D MIPs for slices parallel to the electrode path (x−z view; 50  μm) and perpendicular to the electrode (y−z view, 100  μm). On day 1, apical dendrites in the electrode path appeared bisected, although individual dendrites were still clearly defined and the dendritic tuft at the pial surface remained densely arborized. Three months later, the tissue region immediately above the electrode was absent of apical dendrites and the thickness of the dendritic tuft was dramatically reduced. Dendritic tuft arborization outside of the electrode region and in the window-only animals remained dense and complex and showed clear neurites for the duration of the year-long imaging protocol.

One limitation of these findings is that we did not image animals immediately after window implantation but before electrode implantation; thus there was no true baseline of dendritic organization prior to electrode insertion. Additionally, due to several confounding factors, including changes in the tissue with time, slight differences in the angular rotation of the imaging window relative to the electrode, and electrode movement (measured at the tip of the electrode: mean±SEM: 78.6±17.1  μm; see Fig. S2 in the Supplementary Material), we were unable to identify and track unique dendrite segments over the entire experimental duration.

**Fig. 7:**
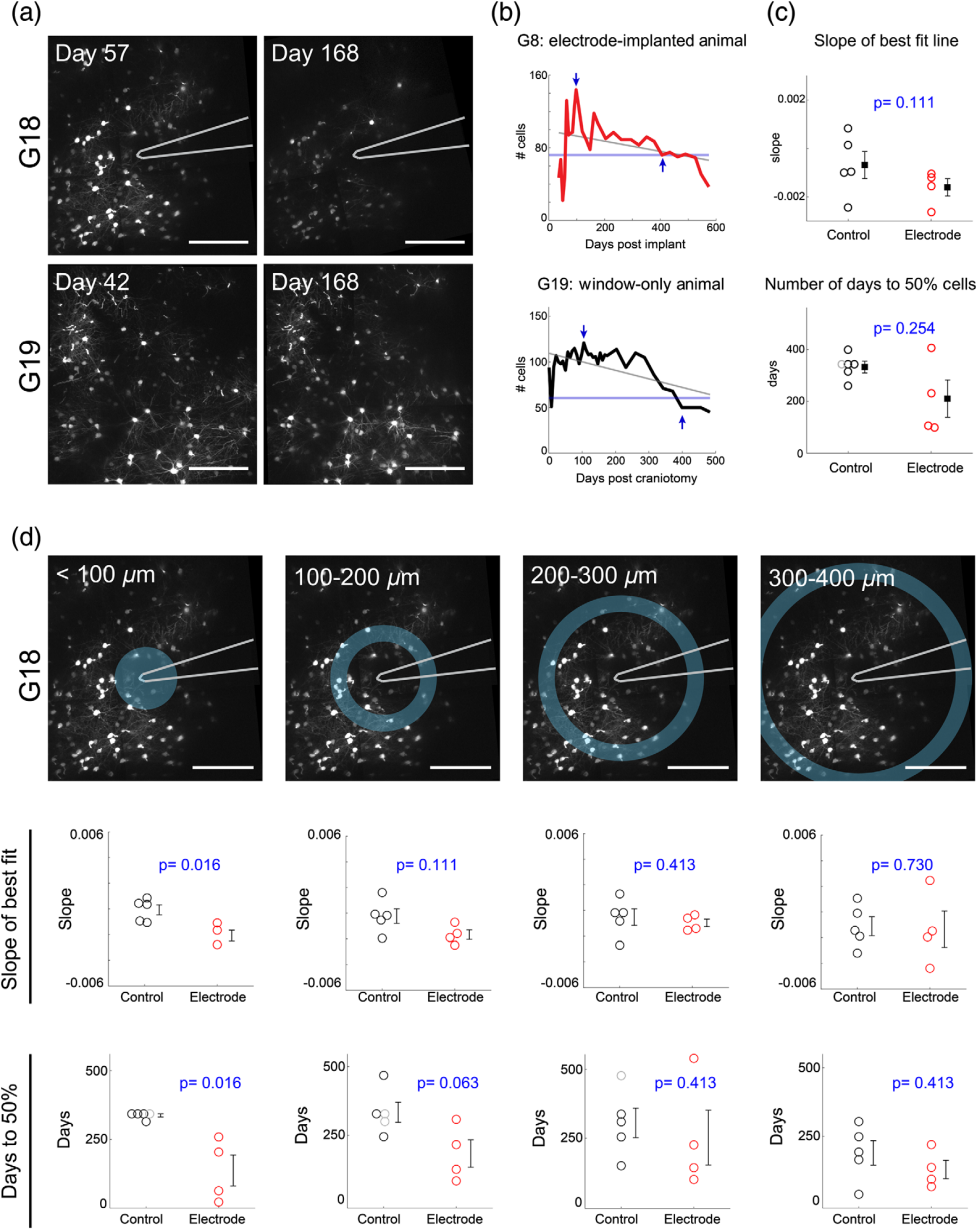
Neuronal loss occurs near the electrode tip. (a) Layer V neurons from an electrode-implanted animal (top) and a window-only animal (bottom) ∼2 months and ∼6 months postsurgery (scale bar=200  μm). (b) Sample traces show loss of neurons over time in an electrode-implanted animal (red) compared with neuronal count in a window-only control (black). Downward-pointing blue arrow denotes maximum cell number, whereas upward-pointing blue arrow denotes point at which cells drop below 50% of max. Linear regression is plotted as a gray line. (c) Animals with electrode implants reached 50% cell loss within 450  μm from the electrode tip by 210.5±72  dpi (error bars show mean±SEM). When not significantly different from the electrode group (Wilcoxon rank sum test, p=0.254), 1 of 5 window-only animals still had not reached 50% cell loss (gray circle). (d) Representative images of regions encompassed by <100-μm, 100- to 200-μm, 200- to 300-μm, and 300- to 400-μm rings. (scale bar=200  μm). Linear regression of neuronal counts over time reveal a significant decrease in cells within 100  μm of an electrode compared with window-only animals (p=0.016) and a slight decrease within 100- to 200-μm distances (n.s.). No decrease is seen at distances >200  μm. Animals with electrode implants reached 50% cell loss within 100  μm from the electrode tip by 137±56  dpi (p=0.016), by 199±50  dpi within 100 to 200-μm distances (n.s.), and by 258±100  dpi within 200 to 300-μm distances (n.s.).

A potential consequence of severed neuronal processes and subsequent dendritic atrophy is cell death. Previous studies have found a significant and progressive reduction in neuronal cell bodies in the immediate vicinity of microelectrode arrays, most noticeably within 100  μm,^15^^,^^37^ although only single postmortem time points were assessed, the latest of which was at four months postimplantation. Here we measured neuronal cell body counts over time in regions surrounding electrodes (Fig. 7). For each animal, we calculated both the slope of the linear regression and the day on which cell counts reached 50% of the earliest maximum value. Due to early superficial vascular changes attributed to the window surgery that partially obscure deep imaging within the first two weeks (discussed in Sec. 3.3 and reported previously in Hammer et al.^22^), the maximum cell count typically did not occur on the first day after implantation. Animals with electrode implants reached 50% cell loss within the full ∼800-×800-μm imaged region by 210.5±72  dpi [Figs. 7(a)–7(c)], but this was not significantly different from window-only animals, which reached 50% cell loss by 332±23  dpi (Wilcoxon rank sum test, p=0.254). Window-only cell counts were more stable compared with electrode-implanted animals within the first 300 days after surgery [Fig. 7(b)], although all animals had evidence of progressive cell loss after 300 days, which could be due to either aging effects or an ongoing neurodegenerative response to the window implantation itself. Motivated by previous studies that reported more localized neuronal loss, we subdivided the full imaging field into four annular ROIs at increasing distances from the electrode tip [Fig. 7(d)]. Animals with electrode implants reached 50% cell loss within 100  μm from the electrode tip by 137±56  dpi (p=0.016) and by 199±50  dpi within 100- to 200-μm distances (p=0.063, n.s.), and linear regression confirmed a progressively decreasing neuronal density closely surrounding electrodes over the entire imaging period. At greater distances from the electrode (200 to 300  μm, 300 to 400  μm), the difference was increasingly less significant; thus long-term neuronal loss primarily occurs within ∼200  μm of the electrode. Since we measured neuronal loss by the lack of YFP fluorescence over time in tracked regions where cells were previously located, we were unable to distinguish whether the progressive absence of cell bodies was attributed directly to cell death or to other causes, such as shadowing from overlying vasculature, growth of glial scar tissue that encapsulates the electrode and can displace surrounding tissue and neurons, or other causes of fluorescence signal loss. Therefore, we cannot definitively rule out the possibility that neurons remain viable despite loss of detected fluorescence signal. Further functional imaging may help validate our interpretation of results. Also, because only layer V pyramidal neurons are labeled with this mouse line, the results are a sparse sampling and do not extend to neurons in other layers and types, such as interneurons.

Together, these results show that neurodegenerative mechanisms, evidenced as dendritic fragmentation and localized neuronal cell loss, precede signs of presumable mechanical/material deterioration of electrodes (saturated LFP and excessive HF noise after 9 months).

### 3.3 Chronic Electrode Implantation and Window Surgery Induce Vascular Changes

The effect of window surgery on superficial (0 to 100 μm) cortical vasculature has previously been characterized by our group.^16^^,^^22^ The window surgery causes a neuroinflammatory response that starts in the first week and extends for several weeks. In that neuroinflammatory period, there is vasodilation in the majority of superficial vessels, which recovers after two weeks in the larger vessels (>50-μm diameter) but can persist in smaller vessels (∼25- to 50-μm diameter). Superficial vascular growth and remodeling accompany this vasodilation as the cortical tissue responds to the window surgery. New superficial vessel growth persists in some animals while in others it is minimal (e.g., Video S1) or abates and returns to the appearance of the superficial vasculature immediately following implantation (Fig. S3 in the Supplementary Material). Importantly, the dynamics of this response are similar for window-only and electrode animals [Fig. S3(c) in the Supplementary Material] and does not appear to affect the deeper capillaries, as long as the surface vessels do not block penetration of the OCT and TPM illumination light. However, it is important to account for this neuroinflammatory response and not interpret changes or differences in the first two weeks as due to electrode implantation.

**Fig. 8:**
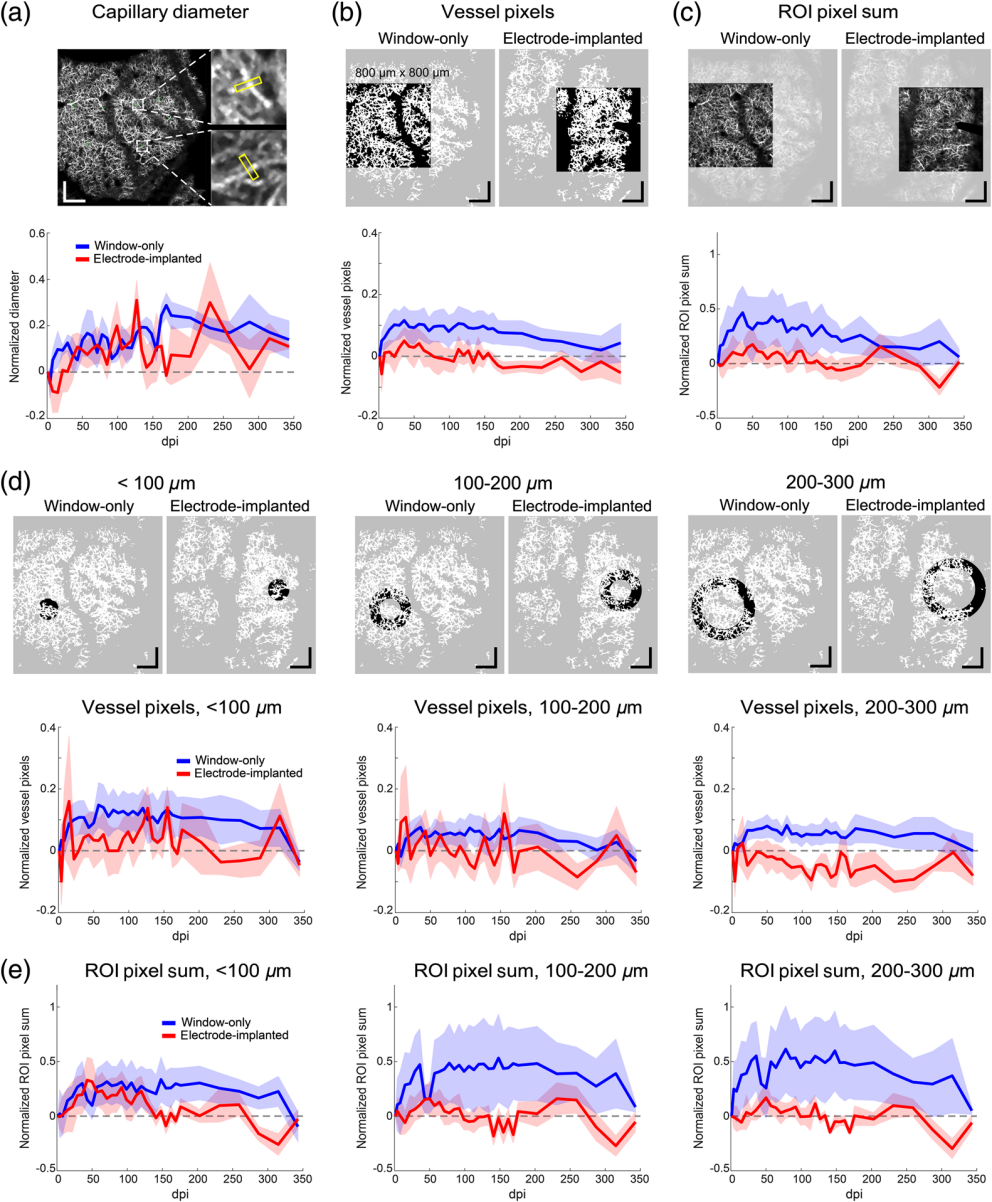
Angiography changes in deep capillaries from window and electrode implantation over 1 year. Each subpanel shows an example of the measurement (top) and the trends for window-only and electrode animals (bottom) over 1 year normalized to 1 dpi. Scale bar in all examples is 200  μm. (a) Average capillary diameter measurements for window-only and electrode animals (two-way ANOVA test, p=0.0015). (b) Capillary density quantified by thresholded vessel pixels within large (800×800  μm) ROI ($p<10^{-14}$). (c) Sum of all gray-scale pixels within large ROI ($p<10^{-10}$). (d) Capillary density (vessel pixels) in annular ROIs near electrode with increasing proximity from electrode tip (ANOVA; <100  μm, $p<10^{-3}$; 100 to 200  μm, p<0.01; 200 to 300  μm, $p<10^{-20}$). (e) ROI pixel sum in annular ROIs (<100  μm, p<0.005; 100 to 200  μm, $p<10^{-9}$; 200 to 300  μm, $p<10^{-13}$). Shaded area for all figures indicates ±SEM.

The deep capillary diameter measurements are shown in Fig. 8(a). The measurements indicate a ∼10% to 20% increase in capillary diameter that persists through the duration of the experiment in window-only animals, consistent with earlier results on smaller superficial vessels. Interestingly, on average, the electrode animals experience some minimal vasoconstriction very early (<21  dpi), followed by a chronic increase similar to the window-only animals. There are also some differences in the late (>150  dpi) response. A two-way ANOVA test revealed a significant difference between electrode and window-only groups (p=0.0015) but no significance among individual time points (p=0.109). Tukey *post hoc* test did not show a significant difference between the two groups at individual time points either. Because the vessel segments were sampled across the entire window, no conclusions can be drawn regarding electrode proximity for capillary diameter. Also the analysis may be slightly biased toward larger capillaries as those were most likely to provide measurements separate from adjacent segments.

The deep capillary density was measured in four ROIs with two metrics as shown in Fig. 8. A relatively large ROI captured any wide-field response [Figs. 8(b) and 8(c)] while the annular regions measured local changes around the electrode tip with decreasing proximity, similar to the neuronal measurements from the previous section [Figs. 8(d) and 8(e)]. The two metrics are “vessel pixels,” which is the total count of thresholded pixels corresponding to capillaries in the ROI, and “ROI pixel sum,” which is the sum of all gray-scale pixel values in the ROI. Video S5 shows the time-lapsed video of the two metrics for one representative (window-only) animal [URL: https://doi.org/10.1117/1.NPh.7.1.015004.5]. The first metric is a measure of pure capillary density, whereas the second is a measure of capillary density and flow. In all ROI analyses for both metrics, except the capillary density immediately around the electrode in the first 150 dpi, the electrode-implanted animals showed no increase or a slight decrease in capillary density and flow. Conversely, the window-only animals showed a persistent increase in capillary density and flow in all regions and across the duration of the experiment. As expected, the capillary density increase [Figs. 8(b) and 8(d)] was slightly lower than the increase in capillary density and flow [Figs. 8(c) and 8(e)], indicating that there was both new capillary growth and an increase in flow in response to the window surgery. In the electrode animals, this response was suppressed. Similar to the capillary diameter measurements, statistical analysis demonstrated that differences between the two animal groups are significant in all four ROIs of both metrics (two-way ANOVA, see p values in Fig. 8 caption), but differences among the individual time points are not significant. Also Tukey *post hoc* analysis showed no significant differences between the two groups at individual time points.

Capillary density changes measured with OCT-A across a cortical patch (e.g., the increased capillary density observed in window-only animals) can arise from new vessel growth (as seen superficially), increased perfusion or reperfusion of existing capillaries, or changes to capillary diameter with changes to flow (vessel dilation). Taken together, the capillary diameter and density measurements in Fig. 8 indicate that diameter changes do not account for the lower density observed in the electrode-implanted animals.

**Fig. 9:**
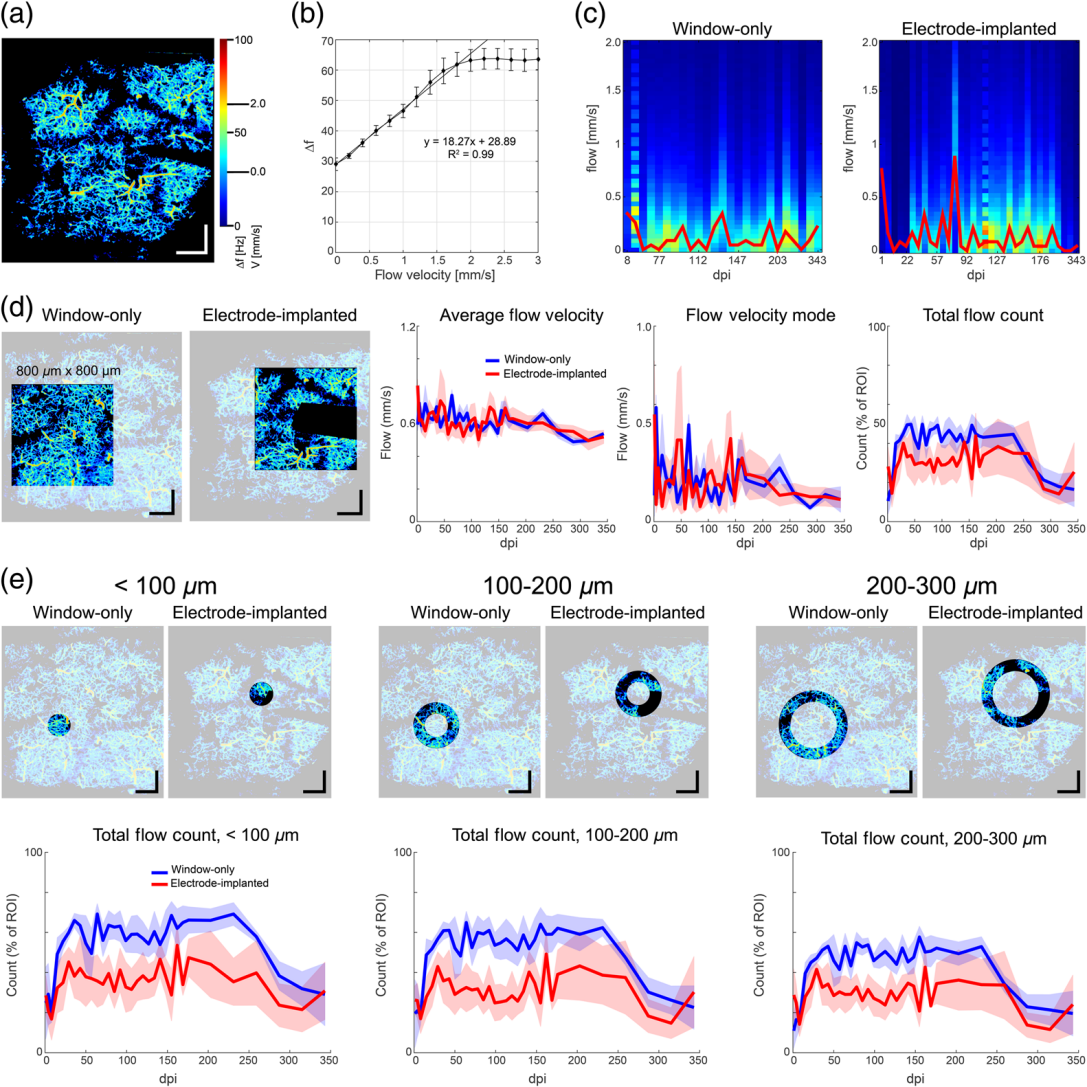
Flow changes in deep capillaries from window and electrode implantation over 1 year. (a) Example flow map from electrode animal showing mapping range (Δf to v) from noise floor to saturation (0 to 2.0  mm/s). (b) Linear calibration curve for bandwidth to velocity mapping calculated from flow maps acquired with a diffuse uniform scattering phantom moving on a precision linear stage. (c) Example histogram maps where intensity corresponds to count in the linear flow velocity range over time. Each vertical line of the map represents a traditional histogram curve. Overlay in red shows the maximum count for each time point in the histogram map. (d) Example large (800×800  μm) ROIs for the window-only and electrode animals. Region over the electrode is masked in further analysis because spurious hyper-reflective signals can arise from electrode pads and other specular surfaces on the electrode. Electrode mask corresponds to entire migratory region over the experiment duration. Metrics collected from the flow maps include average flow velocity, flow velocity mode, and total flow count (see text for description of each). Total flow count is normalized to the total ROI count (excluding electrode mask). There is no statistical difference between the window-only animals and the electrode-implanted animals in average flow velocity (two-way ANOVA, p=0.98) or flow velocity mode (p=0.83), but the difference in total flow count is significant ($p<10^{-5}$). (e) Total flow count in annular ROIs near the electrode with increasing proximity from electrode tip show statistical differences between groups at all distances (two-way ANOVA; <100  μm, $p<10^{-14}$, 100 to 200  μm, $p<10^{-15}$, 200 to 300  μm, $p<10^{-12}$). Scale bar=200  μm on all images. Shaded area for all figures indicates ±SEM.

With capillary velocimetry,^29^ flow velocity can be quantified within the linear range from measurement noise to saturation [Figs. 9(a) and 9(b)], 0 to 2  mm/s for our scan and system parameters. For the flow analysis, videos from over half of the animals taken on the first day were unusable, so normalization was done with respect to the ROI size, rather than a date later than 1 dpi where the initial neuroinflammatory response to surgery could bias the result. While not ideal, the overall starting point and trends shown in Fig. 9 indicate minimal influence from interanimal variability.

The flow velocity maps were further analyzed using the same ROIs used in Fig. 8 for the angiography maps (one square and three annular). Flow velocity over time was best visualized with histogram maps [Fig. 9(c)], showing binned counts as intensities on the flow velocity versus time scales. Because four different ROIs were used and the electrode region was masked, the histogram maps could only be compared if scaled proportionally to the total ROI count. Prior to analyzing the flow velocity maps, pixel values outside of the linear range (i.e., below the noise floor or greater than the saturation value) were excluded from further analysis. Therefore, the analysis excludes regions that have either no flow or very high flow. This bias was necessary to yield an accurate flow velocity analysis and justified in that total flow velocity counts normalized to the total ROI pixels were calculated. This analysis also was relatively immune to surface vessel shadowing because only flow velocity pixels were analyzed. Also little saturation was observed in the flow map videos. For example, in the map shown in Fig. 9(a) with the ROI shown in Fig. 9(d), only ∼1% of flow velocity pixels were above the saturation limit, a value similar to that found in all window-only and electrode animals.

Four metrics were collected from the flow maps [Fig. 9(d)]: the “average flow velocity” (mean flow pixel velocity in mm/s), the “flow velocity mode” (peak count in the histogram), “total flow count” (% of pixels with flow velocity normalized to total ROI pixels), and “total flow velocity” (“average flow velocity” × “total flow count”), the last of which is not shown in Fig. 9. The average flow velocity for both the electrode and window-only animals was ∼0.6  mm/s, which decreased slightly over the experimental duration, either due to animal aging or superficial shadowing from dura/bone regrowth. The most common flow value in the binned histogram data [shown as a red line overlay in Fig. 9(c)] was lower, and no differences were observed between electrode and window-only animals (two-way ANOVA, p=0.98), though both groups showed significant variability from week to week. As expected, the total flow count was low during the neuroinflammatory period and increased initially in the subsequent weeks, followed by a stable period until ∼250  dpi, where aging and surface re-growth caused a late decrease. This temporal dynamic is statistically significant with p=0.0065 using two-way ANOVA. Further one-way ANOVA analysis suggests that this temporal change is significant in window-only animals ($p<10^{-5}$) but not in electrode animals (p=0.998). The divergence in the trend of change is associated with a significant difference in total flow count between the two animal groups (two-way ANOVA, $p<10^{-5}$). However, no statistical significance was revealed between the two groups of animals at individual time points (Tukey *post hoc*). The significant deficits in flow in the electrode animals appeared to be correlated to electrode proximity, where the average difference between electrode and window-only groups was largest for the ROIs near the tip (average delta±SEM: <100  μm=0.191±0.017, $p<10^{-14}$; 100 to 200  μm=0.196±0.020, $p<10^{-15}$), decreased at distances away from the electrode (average delta 200 to 300  μm=0.141±0.019, $p<10^{-12}$), and was smaller for the wide-field ROI (average delta=0.088±0.016, $p<10^{-5}$), where differences in close proximity to the electrode would be muted. Because the average flow velocity was similar for window-only and electrode animals, the total flow velocity adds no further information than that observed in the total flow count and is not shown.

Statistical tests on capillary diameter, density, and flow revealed similar findings, where overall differences between the electrode and window-only groups were significant but differences between the groups at individual time points were not. Given the relatively small sample size and large interanimal variability, the inability to achieve statistical significance at individual time points is not unexpected.

In addition to the systematic changes in angiography and flow from window and electrode implantation shown in Figs. 8 and 9, and Fig. S3 in the Supplementary Material, an idiosyncratic but typically late response to electrode implantation was observed in several animals. Figure S4 in the Supplementary Material and the accompanying Videos S6 and S7 show two such cases [URL: https://doi.org/10.1117/1.NPh.7.1.015004.6 and https://doi.org/10.1117/1.NPh.7.1.015004.7]. In animal G8, a localized flow drop-out occurs at 29 weeks postimplantation (wpi), which appears to partially recover the next month but relapses at later time points. Localized flow drop-out in the vicinity of the electrode was observed by our group previously and may indicate a by-product of gliosis.^23^ Because flow drop-out in OCT-A images manifests as hypo-reflectivity, it cannot be ruled out that flow drop-out is associated with extravasation or hemorrhage and the resultant pooling is responsible for loss of signal. The time-lapsed video from the same animal with merged TPM and OCT images (Video S4) shows concomitant changes in apical dendrite alignment with flow drop-out and localized swelling. In animal G18 at very late time points, capillary stretching (in a direction corresponding to the long-term electrode migration as shown in Fig. S2 in the Supplementary Material) and possible adhesion were observed in the region immediately adjacent to the electrode. The capillary stretching may be due to the electrode hooking a capillary segment during the normal course of its migration. It is not known if any putative capillary adhesion was caused by gliosis or BBB breach. There was no visual evidence of microbleeds or BBB breach in any of the deep capillary time-lapsed videos, though imaging began one day after insertion, so initial insertion effects were not captured.

Vascular measures such as capillary density and flow velocity exhibited relative stability over the chronic implant period, while late decay for time points >1 year probably indicate natural aging. Overall differences between the electrode and window-only groups and localized changes proximate to the electrode indicate that the presence of the electrode influences the tight coupling between the neurons and the capillary beds that supply their metabolic demands.

### 3.4 Glial Responses to Chronic Implanted Electrodes

**Fig. 10:**
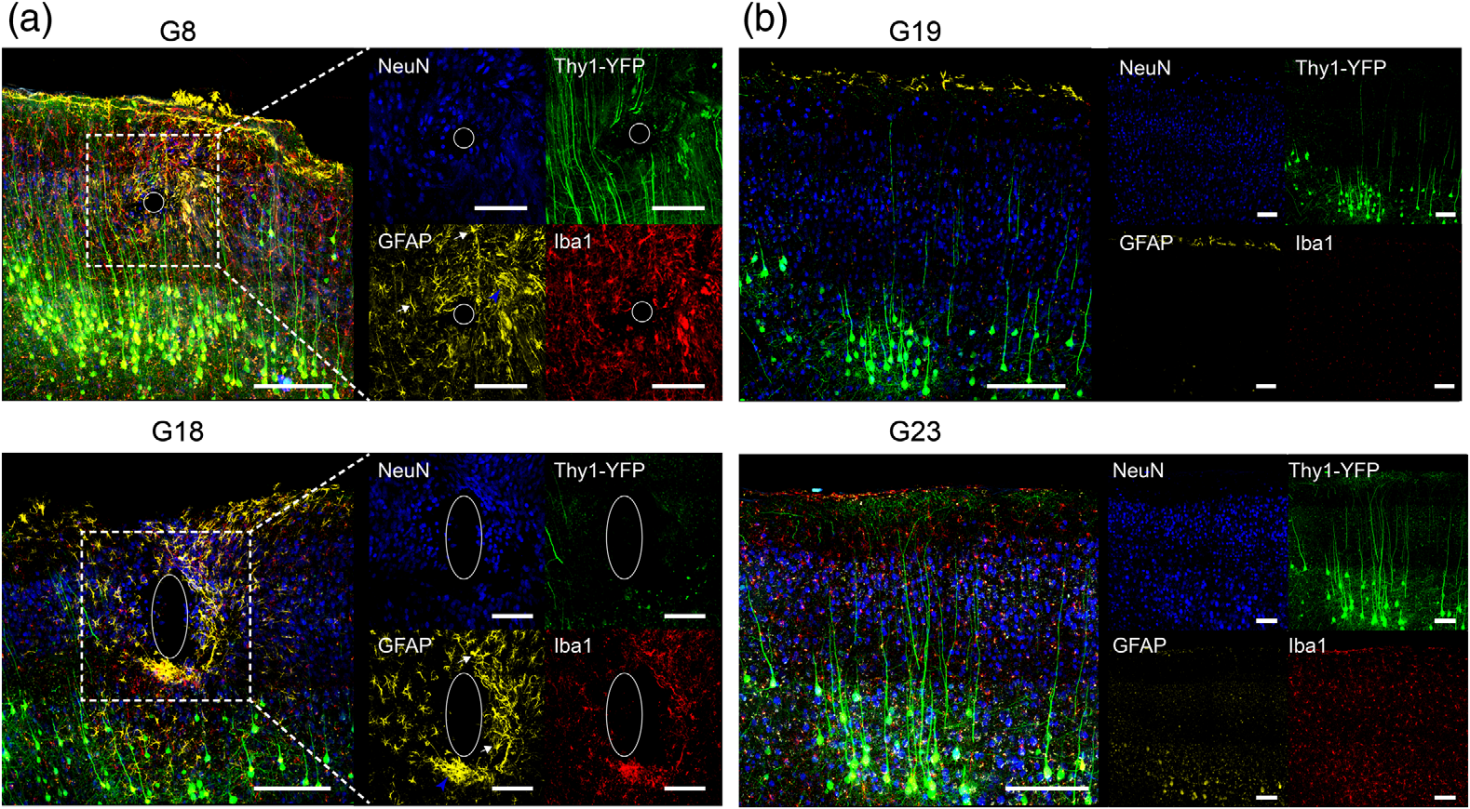
Glial scarring surrounding chronically implanted electrodes. At the culmination of the longitudinal imaging protocol, mice were perfused and stained for neurons (NeuN, blue), astrocytes (GFAP, yellow), and microglia (Iba-1, red). Stained slices from electrode-implanted animals (a) show evidence of extensive glial scarring with increased reactivity of GFAP and Iba-1 in a compact layer around the electrode insertion site (white ovals, G8=586  dpi and G18=491  dpi), whereas stained slices from window-only implanted animals (b) show normal (sparse) distribution of glial cells and neuronal cell bodies (G19=491  dpi and G23=428  dpi). White arrows in magnified GFAP images in (a) indicate hypertrophic astrocytes, and blue arrows show intertwining cells. Transgenic YFP signal in window-only animals (green) shows dendrites of L5 cells radially projecting to the dorsal surface. In electrode-implanted animals, some dendrites appear to curve around the electrode insertion site, whereas others appear severed. (Scale bar for image with merged channels=200  μm, scale bar for individual channels=100  μm).

To evaluate the inflammatory tissue response associated with long-term cortical electrode implantation, glial responses were histologically assessed at the conclusion of the year-long imaging protocol (Fig. 10). Coronal brain sections, cut perpendicular to the electrode path, were stained for neurons (NeuN), astrocytes (GFAP), and microglia (Iba1). The intrinsic transgenic YFP signal in both window-only animals [Fig. 10(b)] and electrode-implanted animals [Fig. 10(a)] shows strong fluorescent labeling of pyramidal neurons, predominantly located in layer V, and the defined structure of apical dendrites radially projecting to the pial surface, consistent with previous mapping studies.^38^ Notably, in the electrode-implanted animals, the dendrites intersecting the electrode path are truncated, and these as well as adjacent dendrites appear to curve around the hollow perforation left by the electrode [Fig. 10(a)].

In healthy cerebral cortex, few astrocytes express detectable GFAP in the middle layers,^39^ and microglial distribution is homogenous with respect to both morphology and distribution.^40^ In our window-only animals, we find low levels of GFAP staining, predominantly restricted to the pial surface and diffuse and dim Iba-1 staining across all cortical layers [Fig. 10(b)]. In contrast, GFAP and Iba1 staining is increased in the entire cortical section of electrode-implanted animals, but it is most concentrated in a tight glial sheath around the electrode. Astrocytes display signs of reactive astrogliosis, including hypertrophied arborization, intertwining processes, and darker GFAP staining. Microglia can be seen to have retracted processes and a denser, more amoeboid morphology characteristic of activated microglia engaged in a foreign body response. Furthermore, G18 in particular shows evidence of electrode movement over time (Fig. S2 in the Supplementary Material), evidenced as two circularly defined regions of empty space. The glial response to an electrode array, particularly substantial glial cell proliferation and device encapsulation, is evident in all of the electrode-implanted animals used in this study and is well documented.^14^^,^^41^

### 3.5 Correlation between TPM, OCT, and Electrophysiology Results

Although TPM is powerful for real-time *in vivo* tissue observation, it is not feasible for implementation in clinical applications. For human use applications, measures extracted from the implanted electrode recordings may serve as biomarkers of tissue change or pathology with proper validation, which may include correlation to imaging results. Therefore, we analyzed the relationship between electrophysiological signals and the imaged tissue response to investigate whether LFP and MUA signals have the potential to predict tissue changes that affect the long-term performance of electrodes.

**Fig. 11:**
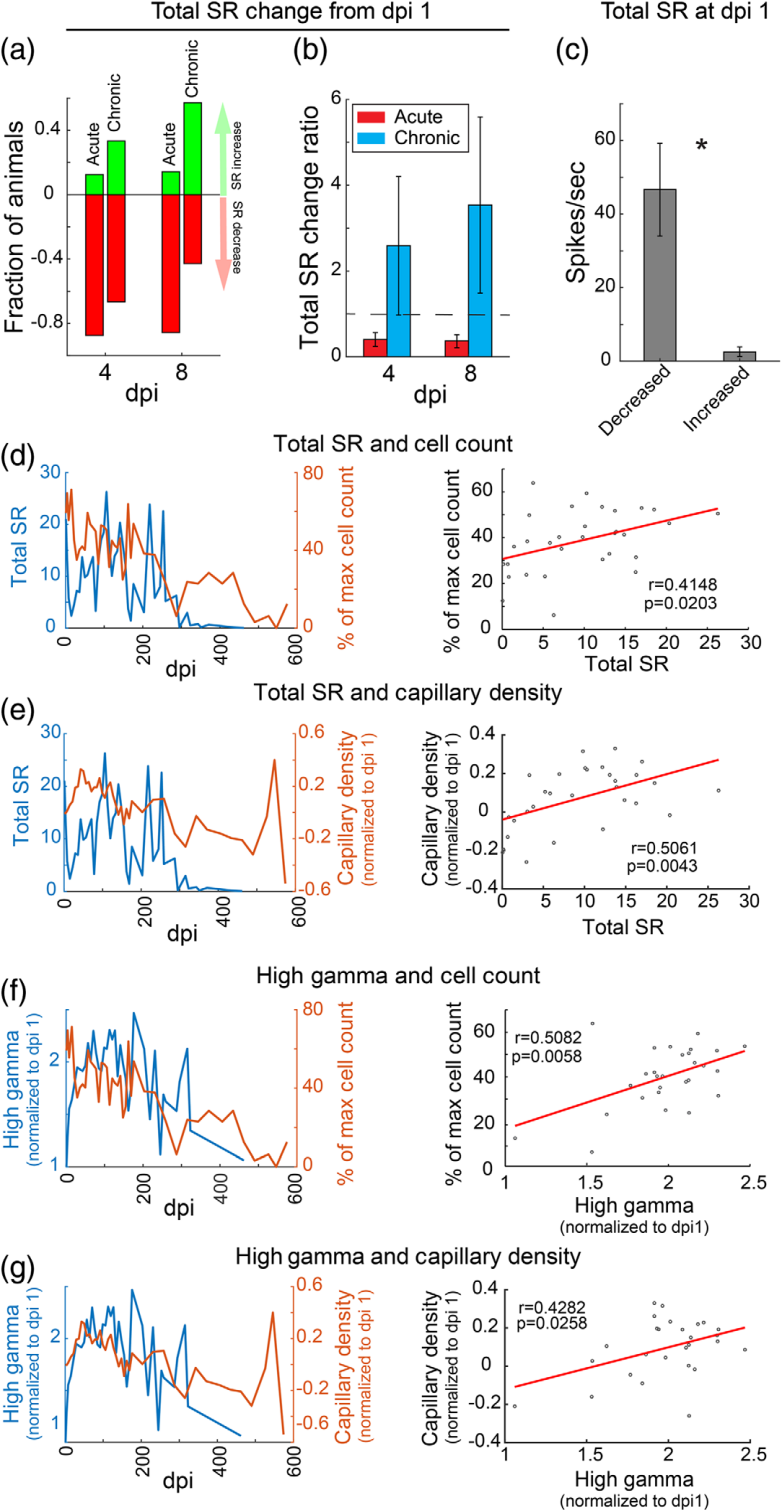
Electrophysiological signals predicting acute and real-time tissue responses. (a) Fraction of animals with acutely reduced or increased tSR in acute and chronic groups, respectively, at 4 and 8 dpi. More animals showed reduced tSR in the acute group. (b) Mean tSR change ratio of acute and chronic groups from 1 dpi to 4 and 8 dpi. (c) Animals with acutely reduced total SR have significantly higher tSR at 1 dpi compared with those with acutely increased tSR. (d)–(g) The significant correlations between electrophysiological data (tSR and high-gamma signal) and tissue responses (percentage of cells relative to maximum number of cells in the field, and ROI pixel sum in angiographs, within 100  μm of electrode) at chronic time points. Left panels show the time course of the two correlated features. Right panels show the Pearson’s correlation between the two features. Only chronic data (>30  dpi) were analyzed for Pearson’s correlation.

To determine whether acute electrophysiological signals can indicate acute adverse tissue responses that prevent long-term data collection, we compared LFP and MUA signals between animals with only short-term data (acute group: n=8), that is, the electrode animals that were acutely sacrificed because a tissue response prevented further imaging, and those with chronic data (chronic group: n=7) within 1-month postimplantation. We did not identify significant differences in LFP and MUA data between the two groups of animals, including power at different frequency bands, tSR, spike amplitude, and SNR, at any acute time points. Neither did we observe significant differences in the acute dynamics of LFP and MUA signals, analyzed by comparing data from 1 dpi with those collected at later time points. However, 7 out of 8 animals in the acute group showed a reduction in SR from 1 to 8 dpi, whereas this occurred in only 3 out of 7 animals in the chronic group [Figs. 11(a) and 11(b)]. Interestingly, animals with decreased tSR had significantly higher tSR at 1 dpi [Fig. 11(c)]. These data suggest that acute tSR and tSR changes within the first week postimplantation may serve as biomarkers for the prediction of tissue changes that prevent long-term use.

To evaluate whether electrophysiological signals can reflect real-time neuronal and vasculature changes, we performed Pearson’s correlation analysis between the electrophysiological signals and the imaged tissue response at chronic time points (>1-month postimplantation) when LFP and MUA signals were relatively stable and consistent among animals (Fig. S5 in the Supplementary Material). Though the degree of correlation in individual animals varies, the mean high-gamma signal and tSR across animals showed a significantly positive correlation with deep capillary density within 100 μm of electrode [Figs. 11(e) and 11(g), and Fig. S5 in the Supplementary Material], and with percent of maximum cell count within 200  μm of electrode [Figs. 11(d) and 11(f), and Fig. S5 in the Supplementary Material]. In addition, noise and spike amplitude is positively correlated with cell count and deep capillary density, particularly within 200  μm of electrode. Interestingly, lower frequency activity (delta to beta) appears to be inversely correlated with deep capillary density and total flow count prominently at regions >100  μm from the electrode. These results imply that a reduction in higher frequency activity, including spikes and high-gamma power, can be a signal of neuronal cell death and deep capillary density decrease immediately around an implanted electrode but is unrelated to local flow. Conversely, an increase in low-frequency activity can indicate a reduction in blood flow at regions further away from the electrode.

In addition to the relationships identified above between chronic electrophysiology data and the imaged tissue response, we found that neuronal viability within 200 μm of electrode showed significantly positive correlation with deep capillary vasculature, including both capillary density and blood flow, in the entire analyzed ROI (0 to 300  μm from electrode tip). Considering the presence of neurovascular coupling, this observation is not surprising. The fact that no apparent microbleeds were generally detected with OCT in the chronic group (except possibly related to the flow dropout shown in animal G8 in Fig. S4 in the Supplementary Material) suggests that neuronal cell death might be one of the factors leading to vasculature changes over time.

## 4 Discussion

The insertion and persistent presence of microelectrodes in cortical tissue induces a response that includes mechanical deformation, BBB breach, gliosis, neuroinflammation, and neurodegeneration.^14^^–^^21^ However, most of what is known about the tissue response to implanted electrodes has been obtained by histological examinations, which are single time-point assessments and provide incomplete information on the temporal dynamics of neuronal and inflammatory activity at the electrode-tissue interface. Thus studies relying on postmortem assessments alone have a limited ability to link structural changes with functional electrode performance. More recently, *in vivo* imaging has been used to study and track tissue and cellular changes over time in animals with implanted electrodes.^14^^,^^16^^,^^22^^–^^25^ Here we used longitudinal *in vivo* experiments in mice with chronic intracortical electrodes to monitor ongoing pathological changes in tissue in real time to fill in gaps in knowledge gained from histology and, importantly, add an investigation of chronic vascular changes to determine how changes in vessel structure and blood flow affect signal quality. We observed progressive localized neuronal cell loss (Fig. 7), transected and atrophying proximal dendrites (Fig. 6), and vascular remodeling and decreased flow velocity (Figs. 8 and 9) in animals with chronically implanted electrodes. More importantly, we demonstrated that electrophysiological signals have the potential to predict tissue responses that affect long-term use (Figs. 4, 5, and 11). Specifically, spike and high-gamma activity reflect proximal neuronal cell viability and capillary density while lower frequency activity implies capillary density and blood flow changes at intermediate regions.

The time-course of the glial response to an electrode array has been fairly well mapped from previous histological analyses^14^^,^^41^ and is consistent with histology results presented here. Electrode insertion triggers an acute inflammatory response involving projection of processes of reactive microglia to the site of insertion as early as one day postimplantation.^17^^,^^26^^,^^42^ As the first-responders to injury, microglia then signal astrocytes through the release of excitatory and inflammatory factors, which respond by proliferating, hypertrophying, and surrounding the foreign body with a dense meshwork of elongated, intertwined processes. Microglia and astrocyte activity plateaus a few months after insertion with complete electrode encapsulation,^14^^,^^17^ but the dense glial scar can last for years.^39^ The presumed function of scar-forming astrocytes is to segregate healthy functioning tissue from diseased tissue or a foreign object,^39^ although in doing so, the tight junctions between cells that make up the sheath prevent the diffusion of ions and neurotransmitters, thus progressively insulating the electrode from local neurons and leading to reduced signal quality.^14^^,^^43^^–^^45^ However, other studies have challenged the supposition that the glial sheath on its own is responsible for degrading signal quality, as evidenced by modeling results^46^ and studies that successfully continued to record signals even after scar formation.^12^^,^^47^ In this study, the glial response was only assessed at a single time point at the culmination of the imaging protocol, at which point all electrode-implanted animals showed evidence of astrogliosis and activated microglia concentrated in a tight glial sheath around the tissue perforation left by the electrode. Notably, electrodes from each of these animals stopped recording analyzable neuronal signals at about a year postimplantation. Future studies that take advantage of double- or even triple-colored glial cell transgenic mouse lines^48^ can more precisely delineate the progression of the neuroinflammatory response to electrode presence as we have done here specifically for neurons.

Histological evidence has also shown a loss of neuronal cell bodies within two weeks of implantation^37^ and a progressive loss of dendritic processes as early as four months.^15^^,^^17^ Previous studies have hypothesized that the initial mechanical trauma of electrode implantation and resulting neuronal and neurite distortion causes a cascade of events that ultimately result in neurodegeneration and cell death, in a process that may be similar to a traumatic brain injury.^25^^,^^49^ One of the earliest molecular effects of this mechanical distortion is an immediate and prolonged increase in calcium concentration in neurons adjacent to the electrode, which can result in excitotoxic cell death and neurite degeneration.^25^^,^^49^ To support this finding, studies performing targeted dendrotomy using laser pulses have found that severing the apical trunk of layer V pyramidal cells enhances the excitability of these cells by lowering the threshold for action potential generation and increasing spike amplitude.^50^^,^^51^ The increased excitability eventually leads to rapid injury-induced degeneration and clearance of dendrite fragments distal to the severed site, similar to how axons are cleared by a process known as Wallerian degeneration after being severed from the cell body.^50^^–^^52^ This is in line with our observation that tSR at 1 dpi appears inversely correlated with imaging longevity, which is related to the acute adverse tissue response. In addition, we observed transected apical dendrites on the first day postimplantation and neurite fragmentation as early as 3 months later (Fig. 3). There was also evidence of mechanical distortion in the y−z cross section of TPM images, which was perhaps even more evident in the histological sections, with “bent” apical dendrites adjacent to the electrode path.

It is certainly true that every craniotomy and microelectrode insertion causes some form of BBB breach and release of proinflammatory cytokines, reactive oxygen species, and nonresident cells, in particular, circulating leukocytes and macrophages, which lead to a neuroinflammatory cascade.^16^^,^^17^^,^^37^ The severity of the response appears to depend on a number of parameters related to the dimensions of the microelectrode and the initial implantation trauma, although studies using stab wound—electrode insertion and quick retrieval—as a control for the acute injury have revealed limited to no glial reactivity and neuronal loss in response to this procedure without permanent electrode presence.^15^^,^^17^ Still, given the variability in surgical implantation of electrodes, tissue changes are idiosyncratic and difficult to reproduce across implants, both within the study presented here and in previous investigations.^25^^,^^26^

A critical element influencing tissue response severity to electrode implantation is whether puncturing major vessels and extravasation is avoided,^14^^,^^23^^,^^53^ not to mention the difficulty in avoiding microhemorrhages.^26^ Using OCT angiography, we previously observed that the craniotomy and window implantation surgery itself is traumatic and elicits a neurovascular response, particularly in the superficial layers (up to ∼100  μm) below the window.^22^^,^^23^ If major vessels are avoided during insertion, the deeper capillary network surrounding the electrode remains intact.^23^ However, this neurovascular response may more closely model clinical conditions than other preparations (e.g., thinned skull without craniotomy) for procedures that involve implanted electrodes. In this study, we consistently saw vascular growth in the superficial layer, either in the dura or pia mater, though the growth varied from animal to animal (Fig. S3 in the Supplementary Material). The dura and its associated vessels are densely innervated by nociceptive nerves, which have high mechanosensitivity.^54^ The direct contact of the dura with the cover glass, which is a hard, foreign body, may induce hyper-reactivity in the dura enough to cause inflammatory responses and vascular regrowth. Drew et al.^55^ also found greater microglial activity in mice with cranial windows compared with the polished reinforced thinned skull preparation. In the analysis and results, we sought to separate the effects of the window surgery from those of the inserted electrode. This was done primarily spatially, where the examined tissue and observations were axially confined to the deeper layers near the electrode tip, and when the effects correspond laterally to the region around the electrodes.

The vascular response to electrode presence includes local flow drop-out and deficits in total flow, suppressed capillary growth, and mechanical adhesion and strain (Figs. 8 and 9, and Fig. S4 in the Supplementary Material). The time-course for these events was variable, but in general, the deficits in total flow were evident within a few weeks postsurgery and persisted chronically, the localized flow drop-out occurred at later time-points (but can occur within hours^23^), and adhesion of electrodes to capillaries developed late (many months after insertion). The local flow drop-out in our earlier acute study was transient,^23^ whereas in this chronic study it was multiphasic—partially recovering after the first instance but then reoccurring. This flow drop-out may be evidence of BBB disruption, though this was not directly investigated. Because OCT-A derives contrast from flowing erythrocytes, hyporeflectivity in OCT-A images—what we refer to as “flow drop-out” in this and previous papers^23^—is ambiguous in terms of the precise microscopic capillary changes that are occurring. It may indicate hypoxia (via loss of flow and reduced perfusion), or it could indicate capillary breach, hemorrhage, and pooling of static blood, which is, in the short-term, reabsorbed. The decrease in flow was greater closer to the tip. Conversely, the suppression in capillary growth was most pronounced at further distances from the electrode tip, although changes within 100  μm were also observed at chronic time points (after 150 dpi). The interplay between capillary density and flow in the immediate vicinity of the electrode thus warrants further investigation.

While structural studies with multimodal imaging such as the one presented here provide an exceptional amount of information of the dynamics of the response to implanted cortical microelectrodes, only correlating the structural results to functional measures will provide evidence of the precise causes of failure. Here we have established the real-time relationship between electrophysiology and tissue damage by comparing changes in LFP and MUA recordings to neuronal cell loss and vascular effects of implantation. We find that the progressive high-gamma oscillation and SR reduction both correlated positively with localized cell loss and decreasing capillary density near the electrode, indicating that the deteriorating signal quality observed may signal neuronal and vascular damage. We also identified an interesting inverse correlation between SR on the first day postimplantation and acute SR change within the first week, suggesting that immediate postimplantation recording quality may predict tissue damage and long-term fidelity of the electrode. Although TPM is mostly confined to the preclinical research arena owing to the relative cost of ultrafast lasers and limitations in imaging depth, quantitative electrophysiological recordings can be acquired with the same device implanted for stimulation in a clinical setting and can be used as a diagnostic tool for the detection of neural injury both acutely and chronically. The correlations we identified between electrophysiological changes and neuronal and vascular degeneration can inform clinical recordings by relating degrading signal quality to the underlying tissue changes on a cellular level.

Ultimately, the goal of this and similar studies is not only to increase our understanding of the causes of electrophysiological device signal loss, especially using the advantages that *in vivo* imaging provides, but also to develop strategies and approaches to improve chronic device reliability. Kozai et al.^14^ offered several areas of exploration, including use of biomimetic coatings, optimization of implant parameters (flexibility, size, and shape), and personalized vascular mapping in the presurgical planning phase. In terms of this last approach, multimodal imaging, and especially OCT-A, may be especially well-suited to the task. In surgical neurodevice implantation in humans, a craniotomy is performed, so there is no requirement to achieve (relatively) deep tissue penetration or more accurately, light penetration through the skull. Unlike TPM, which as previously discussed is not clinically feasible, OCT has already achieved clinical translation for ophthalmology, and one recent advance is integration into surgical microscopes.^56^ Finally, OCT-A has several fundamental characteristics that recommend it for a presurgical neurovascular mapping application. These include acquisition of angiography images without dyes, the natural mesoscopic scale suited to OCT, and the ability to detect flow in both penetrating arterioles and capillaries and provide direct measures of vessel oxygen delivery, load, and consumption.^57^ As such, OCT-A may be an excellent candidate for image-guided neurological device insertions to minimize trauma and BBB disruption.

Differences in neuroanatomy and neuroimmune response between species need to be considered when translating the interpretation of our results in transgenic mice to human subjects. Unfortunately, histological characterization of temporal dynamics of biological responses to intracortical electrodes in humans is not feasible. A short-term study of tissue responses to subdural electrodes in epilepsy patients suggests similar meningeal and perivascular immune responses to findings from rodents.^58^^,^^59^ Studies to investigate blood-based GFAP expression after a traumatic brain injury also show similar acute responses between humans and rats.^60^^,^^61^ These studies indicate a general, systemic similarity of neurological responses to injury between species.

In summary, our longitudinal, multimodal study characterizes the time course of neuronal, vascular, and physiological changes that occur in response to chronic cortical electrodes and identifies the relationship between the biological tissue response and deterioration in signal quality. Future studies incorporating advanced transgenic mouse models with multilabeled fluorescent cells (Cx3cr1, GFAP, Prism) or activity-dependent expression (GCaMP calcium imaging) and simultaneous electrophysiological recording and stimulation will be essential to further characterizing the neuroinflammatory sequelae following microelectrode insertion and electrode stimulation and investigating relationships between various structural and functional outcomes. This integrated approach to elucidate the complex structure–function relationship associated with BCI devices is currently underway in our laboratory.
