# Supplementary material for: Longitudinal multimodal assessment of neurodegeneration and vascular remodeling correlated with signal degradation in chronic cortical silicon microelectrodes
Source: Neurophotonics. 2020 Jan 30;7(1):015004. doi: 10.1117/1.NPh.7.1.015004 (PMC6991888; doi:10.1117/1.NPh.7.1.015004)
Supplement: Supplementary file 1 [file NPh_007_015004_SD001.pdf]

Supplemental Material for

Longitudinal multimodal assessment of neurodegeneration and vascular remodeling correlated with signal degradation in chronic cortical silicon microelectrodes

Krystyna Solarana[1,†], Meijun Ye[1,†], Yu-Rong Gao[1,2], Harmain Rafi[1], Daniel X. Hammer[1,]*

[1]Division of Biomedical Physics, Office of Science and Engineering Laboratories, Center for Radiological Devices, Food and Drug Administration, 10903 New Hampshire Avenue, Silver Spring MD 20993, United States
[2]Current affiliation: Department of Neuroscience and Multiphoton Imaging Core Facility, University of Rochester Medical Center, Rochester NY 14642, United States

Corresponding author: *daniel.hammer@fda.hhs.gov
[†]**Co-first authors**

Declarations of conflicts of interest: No conflicts of interest, financial or otherwise, are declared by the authors.

This PDF file includes:

Figures S1 to S5
Captions and Representative Frames for Supplemental Videos 1 to 7

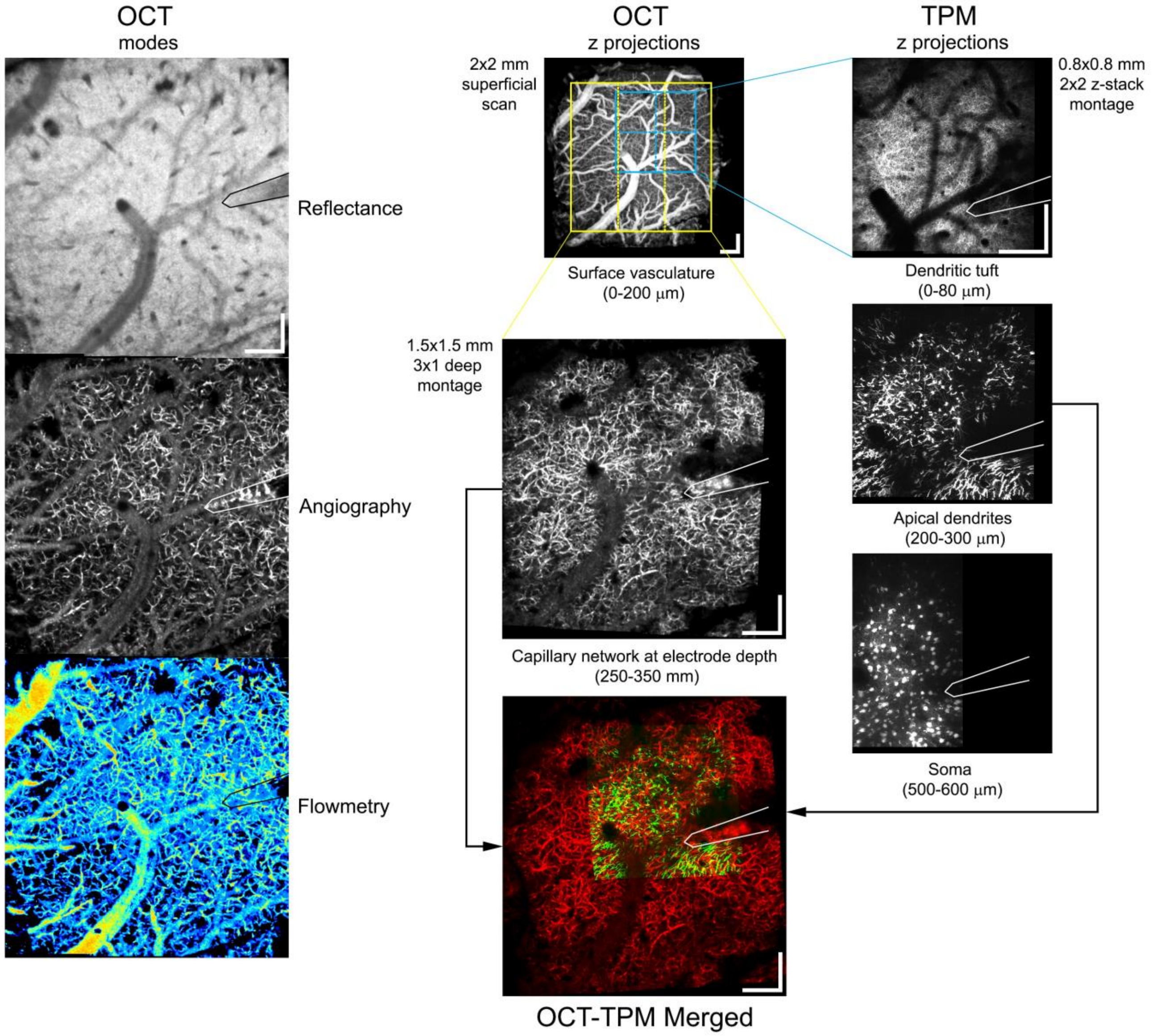

Supplemental Figure 1: OCT-TPM multimodal imaging provides cellular-level visualization of tissue changes in the region adjacent electrode. OCT output modes from volumetric scans include reflectance, angiography, and flowmetry maps. *En-face* views at the depth of the electrode are shown. The 2×2 mm superficial scan of surface vasculature is used to guide deeper OCT (3×1) and TPM (2×2) montages of capillaries and layer V neurons (yellow and cyan overlays, respectively). The TPM z-stack montage through cortical depth shows the superficial dendritic tuft, apical dendrites, and deeper cell soma. The OCT capillary map and TPM dendrites at approximately the same depth can be merged into a false color representation of the region and also viewed in time-lapse across the experimental duration (**Supplemental Video 4**). Deep vascular and neuronal images are MIP. OCT reflectance and superficial vascular and neuronal images are AIP. Electrode location indicated in each panel. Scale bar in all panels is 200 μm.

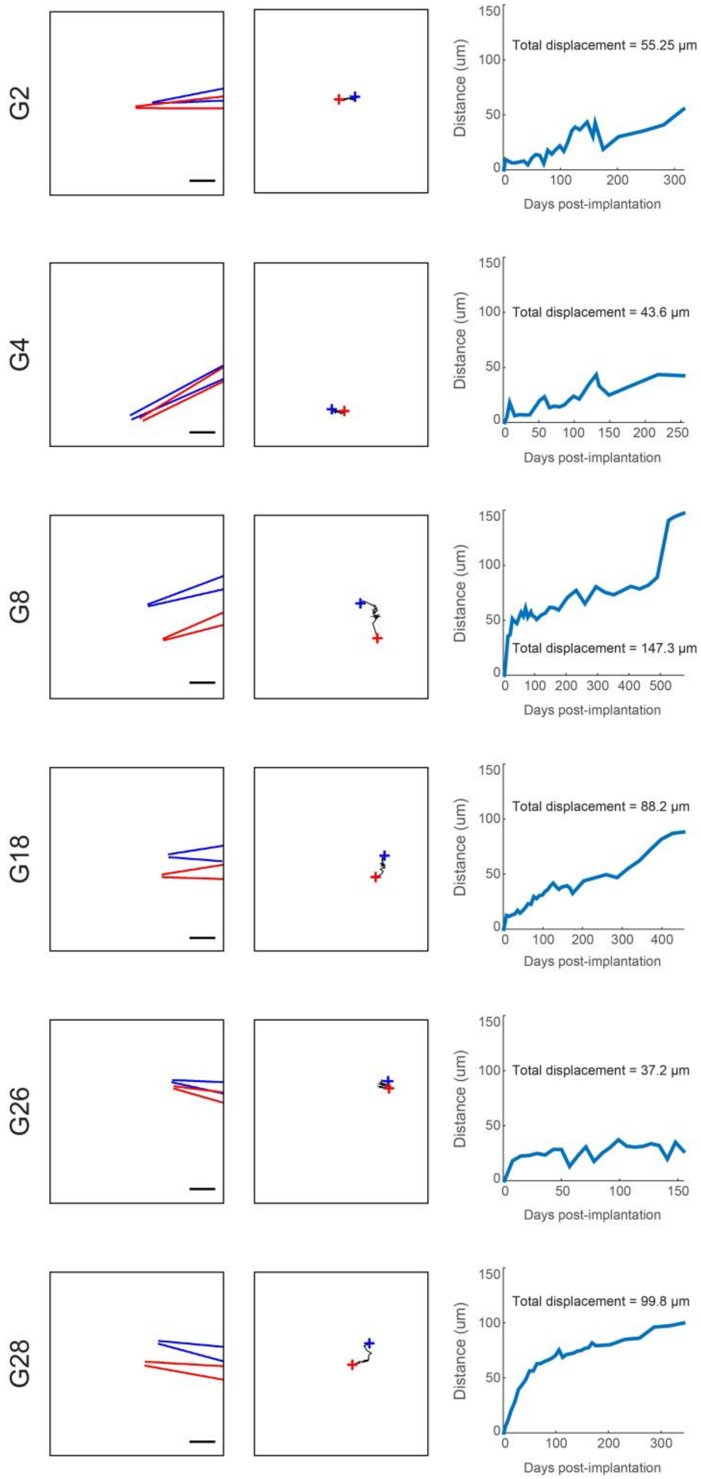

Supplemental Figure 2: Slow migration of electrodes through cortical tissue over time. *En-face* (*x-y* plane) OCT-A images (in which the profile of the electrode is clearly visible) from each imaging day were registered using ImageJ's StackReg plugin and the tip of the electrode was manually selected in each image. The initial position of the electrode is shown as a blue outline for each electrode-implanted animal, and the final position is shown in red (*first column*). The middle column shows the starting (blue) and final (red) locations as well as the drift trajectory of the electrode as a black line. The third column plots distance of the drift as a function of time. On average, electrodes moved 78.6 ± 17.1 μm (mean ± SEM) as measured at the tip over the course of the chronic imaging experiment.

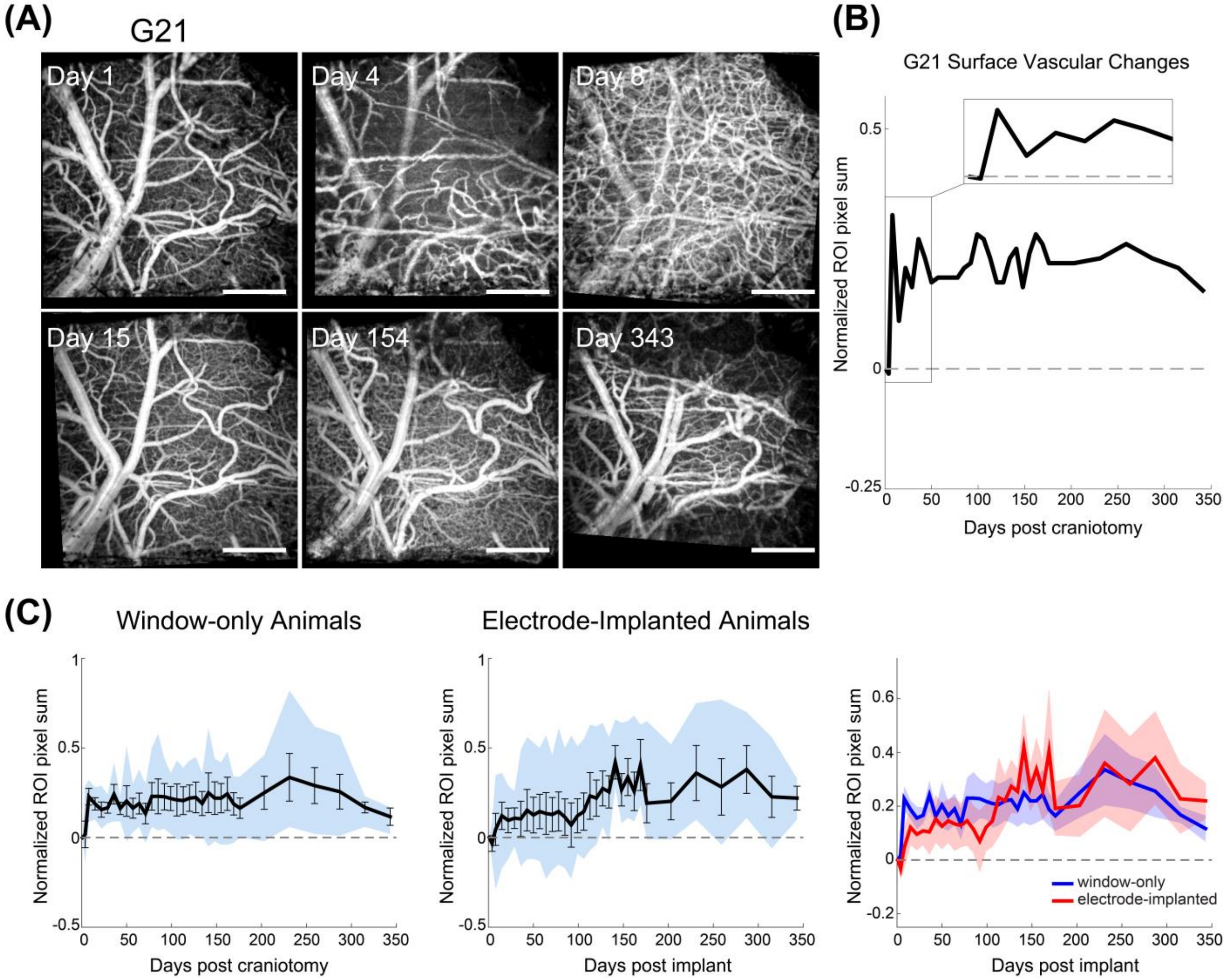

Supplemental Figure 3: Surface (pial) vascular changes are due to window surgery in window-only and electrode animals. (**A**) *En-face* images of long-term surface vascular changes show a typical sequence in the first year post implantation. One day after surgery the vessels appear normal, but by 4 dpi inflammation and edema obscures all but the most superficial vessels. Rapid neovascularization follows (8 dpi) and subsides after the first two weeks (15 dpi), with a slight increase in vascularization compared to 1 dpi. Surface vessels are typically stable over the rest of the year, with common dura/bone regrowth (154 and 343 dpi) beginning near the edges of the imaging window. By the end of the first year, pial vasculature can also become disorganized (343 dpi). Scale bar = 500 µm. (**B**) Prototypical curve depicting changes in surface vessel coverage from the animal depicted in (**A**) using the normalized ROI (nominal size: 250 × 250 pixels, 1 × 1mm) pixel intensity sum from the angiograms. Data is normalized to 1 dpi. Inset shows vascular changes from 1 to 50 dpi at a greater resolution. (**C**) Plots show normalized ROI pixel intensity sum (thick line is group average, bars show standard error, minimum and maximum denoted as shaded blue area) from the angiograms for the window-only and electrode groups. Third panel shows overlayed averages for window-only animals (blue) and electrode-implanted animals (red) with shaded regions for each indicating standard error. All data is normalized to 1 dpi.

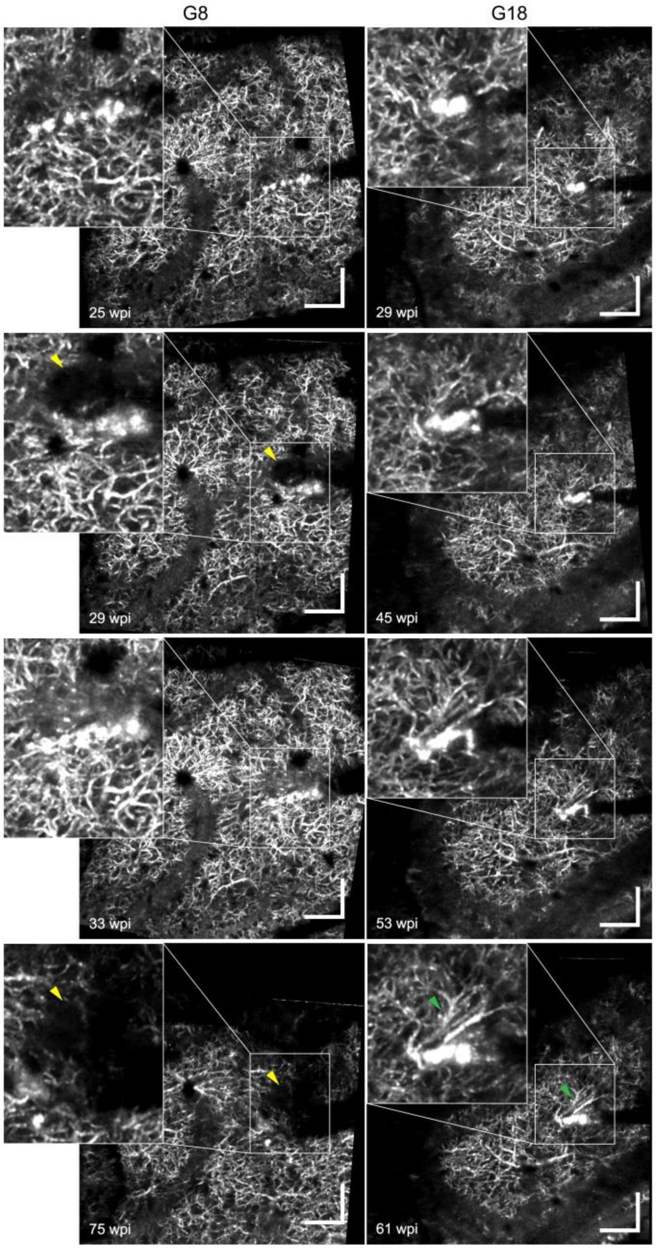

Supplemental Figure 4: Long-term changes around electrode visualized with OCT-A. Temporal sequences in two electrode animals where changes were observed in a spatially confined region around the electrode. Accompanying videos show full sequence across the lifetime of the animal and better visualization of change. In each figure panel and video frame, the capillaries near the depth of the electrode are shown (MIP, stack thickness = 200 µm) as well as a ROI near the electrode zoomed by 2×. In one animal (G8, left panels), an edematous region develops adjacent to the electrode at 29 wpi (yellow arrowhead). Note the swelling concomitant with localized flow drop-out as observed in the outward movement of adjacent capillaries from the electrode in the video. The region partially recovers by 33 wpi but never fully recovers and all timepoints imaged after 42 wpi show a hyperreflective region without capillary flow (yellow arrowhead). In another animal (G18, right panel), electrode movement in later timepoints causes mechanical stretching of the capillaries. At 29 wpi, the region around the electrode appears normal. Stretching starts around 45 wpi and persists through 69 wpi (green arrowhead). The capillaries adjacent to the electrode are elongated in the direction parallel to the line of movement. It is not clear if there is any adherence between the electrode and capillaries caused by gliosis or clotted blood (BBB breach). Scale bar = 200 µm.

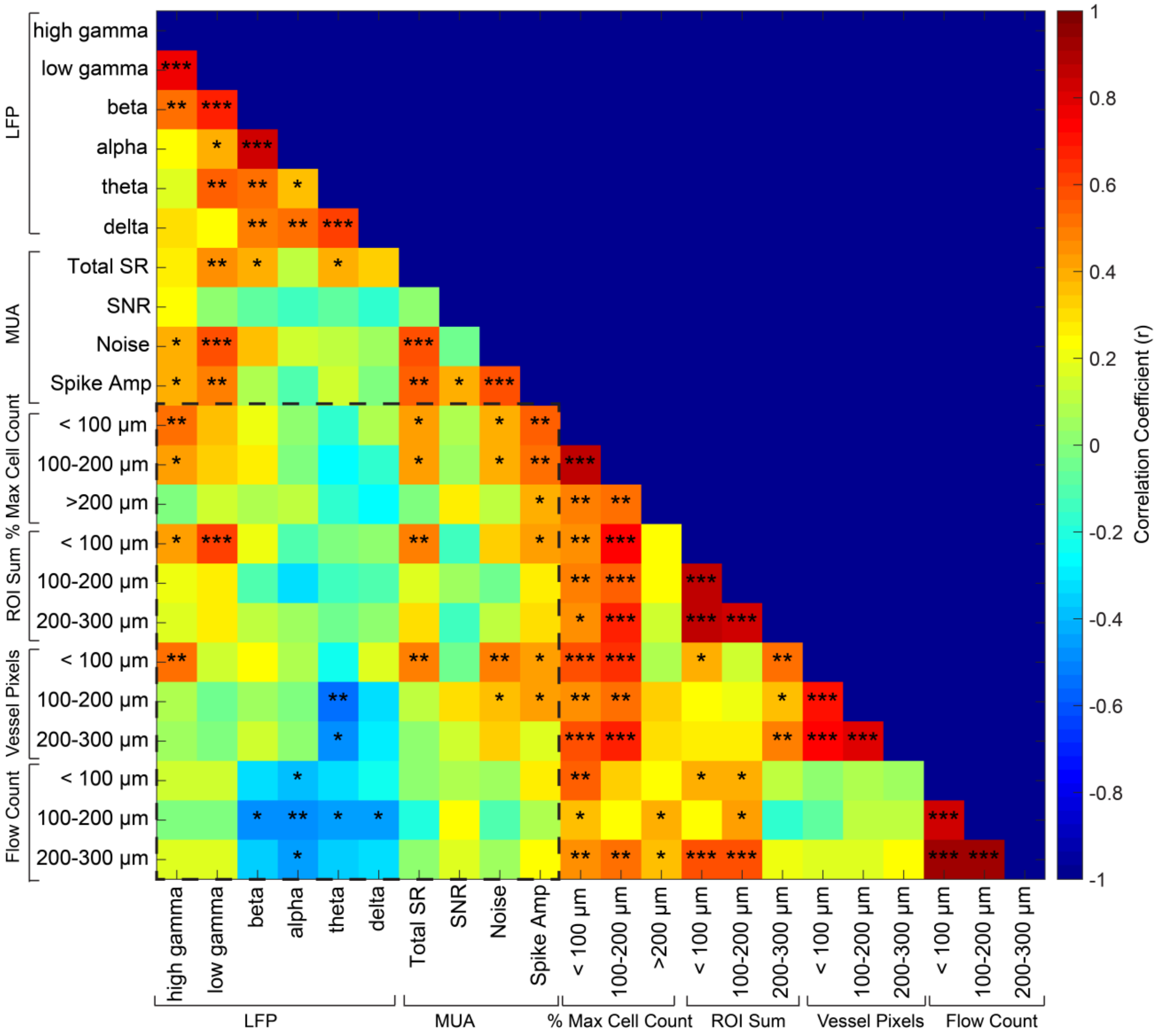

Supplemental Figure 5. Pearson's correlation between OCT, TPM, and electrophysiology results in pairwise. Dotted box indicates correlations between electrophysiology data and tissue responses. (* p<0.05, ** p<0.01, *** p<0.001).

Supplemental Videos (Links to the supplemental videos are provided in the main text.)

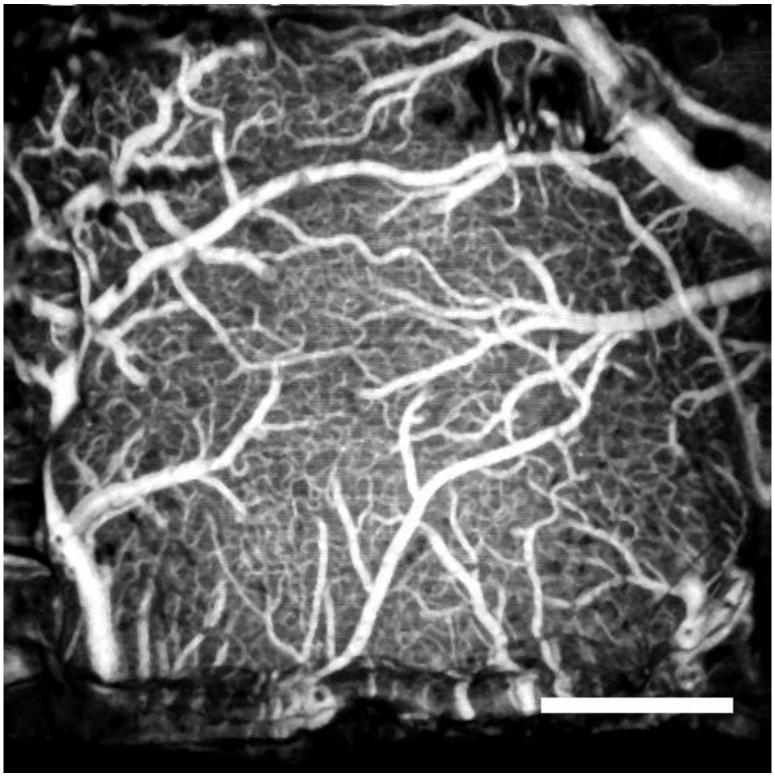

Supplemental Video 1: Example time-lapsed surface (pial) vascular map from OCT-A AIP projections in a window-only animal in the region up to 200 µm below the cranial window. Note the neuroinflammatory response in the first two weeks (mild in this animal) and the late dura/bone re-growth that eclipses light penetration preventing further imaging in those regions. Scale-bar = 500 µm.

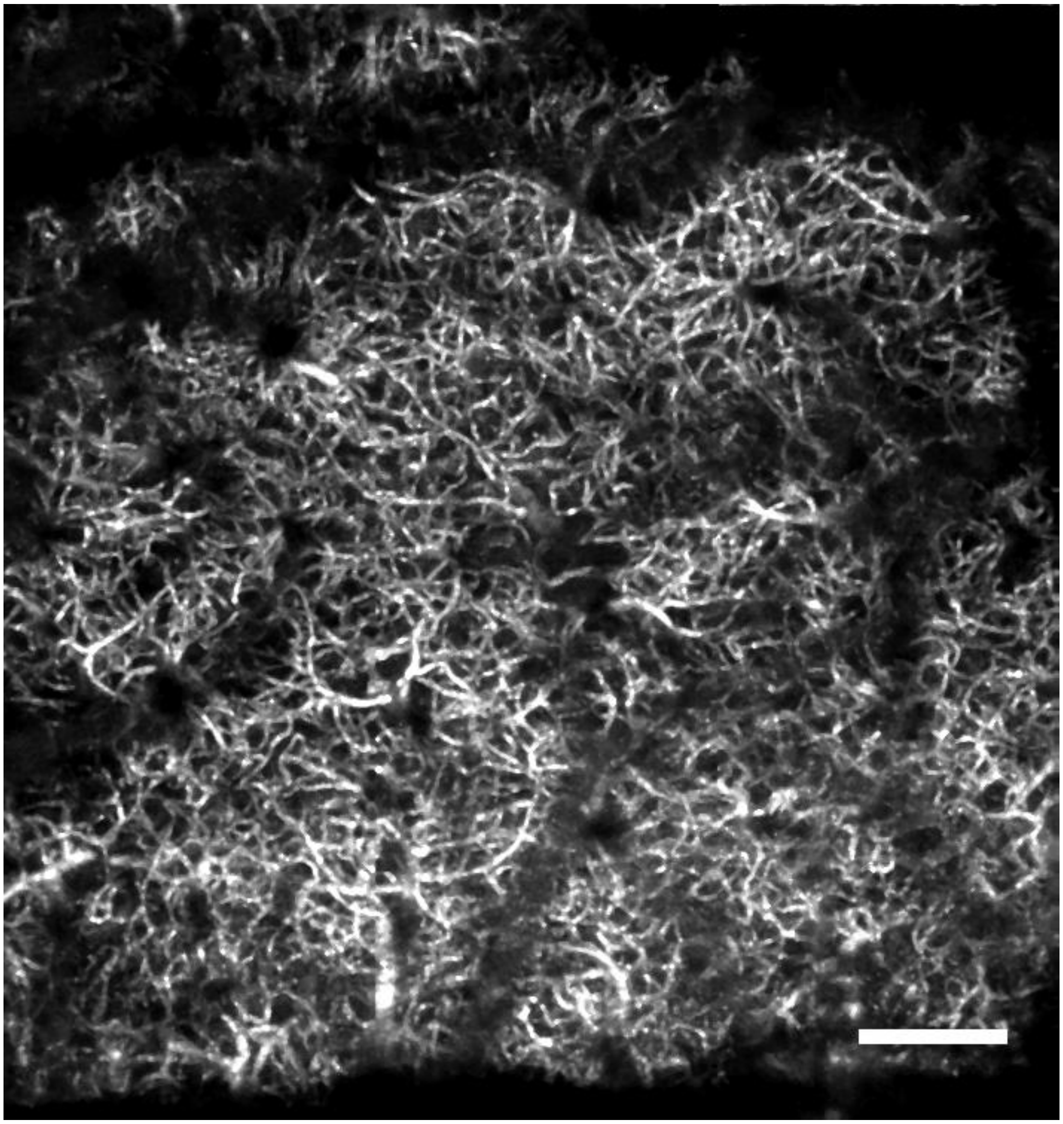

Supplemental Video 2: Example time-lapsed deep capillary vascular map from OCT-A MIP projections in the region ~300-400 µm below the window in the same window-only animal as **Supplemental Video 1**. Scale-bar = 200 µm.

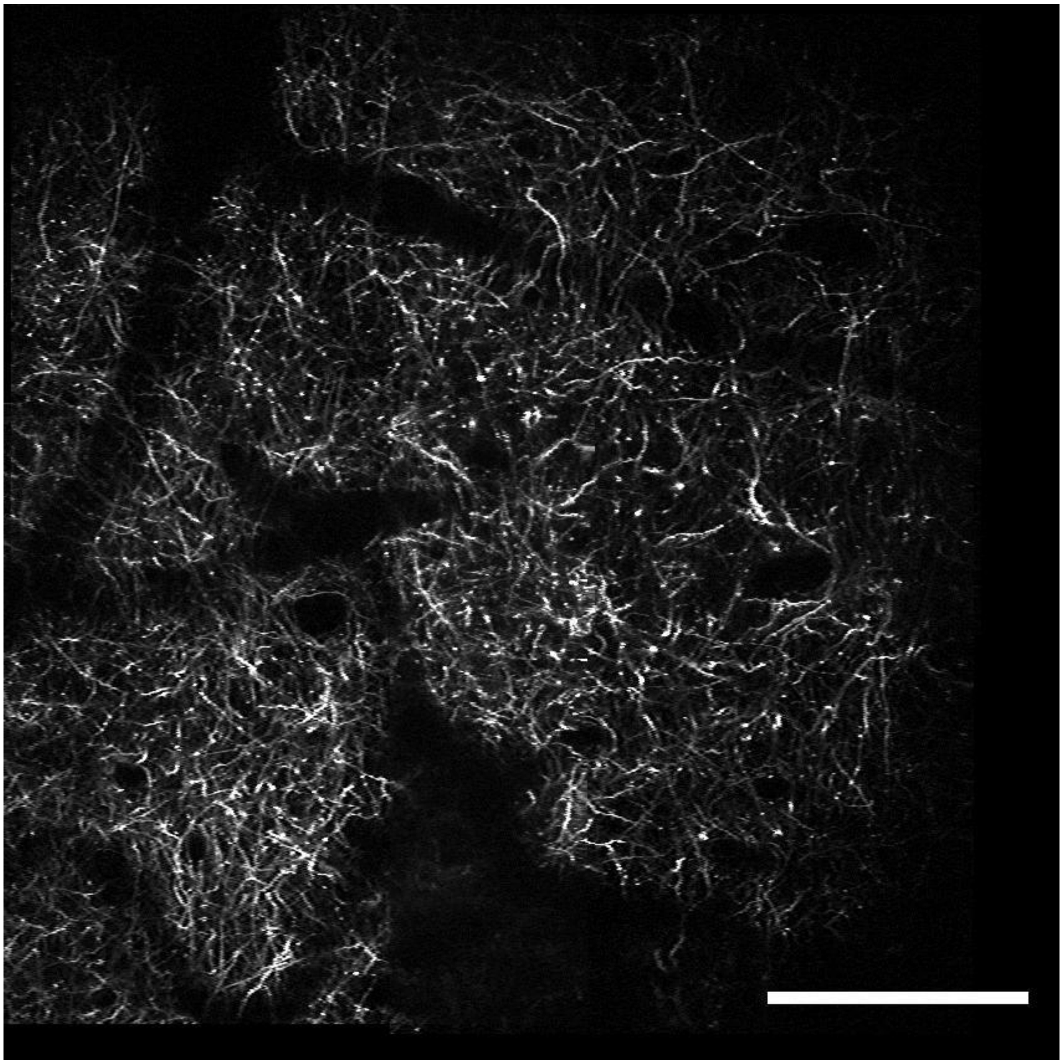

Supplemental Video 3: Example TPM *z*-stack fly-through in an electrode-implanted animal (G28) from the dendritic tuft to the layer V pyramidal neurons. Scale-bar = 200 µm.

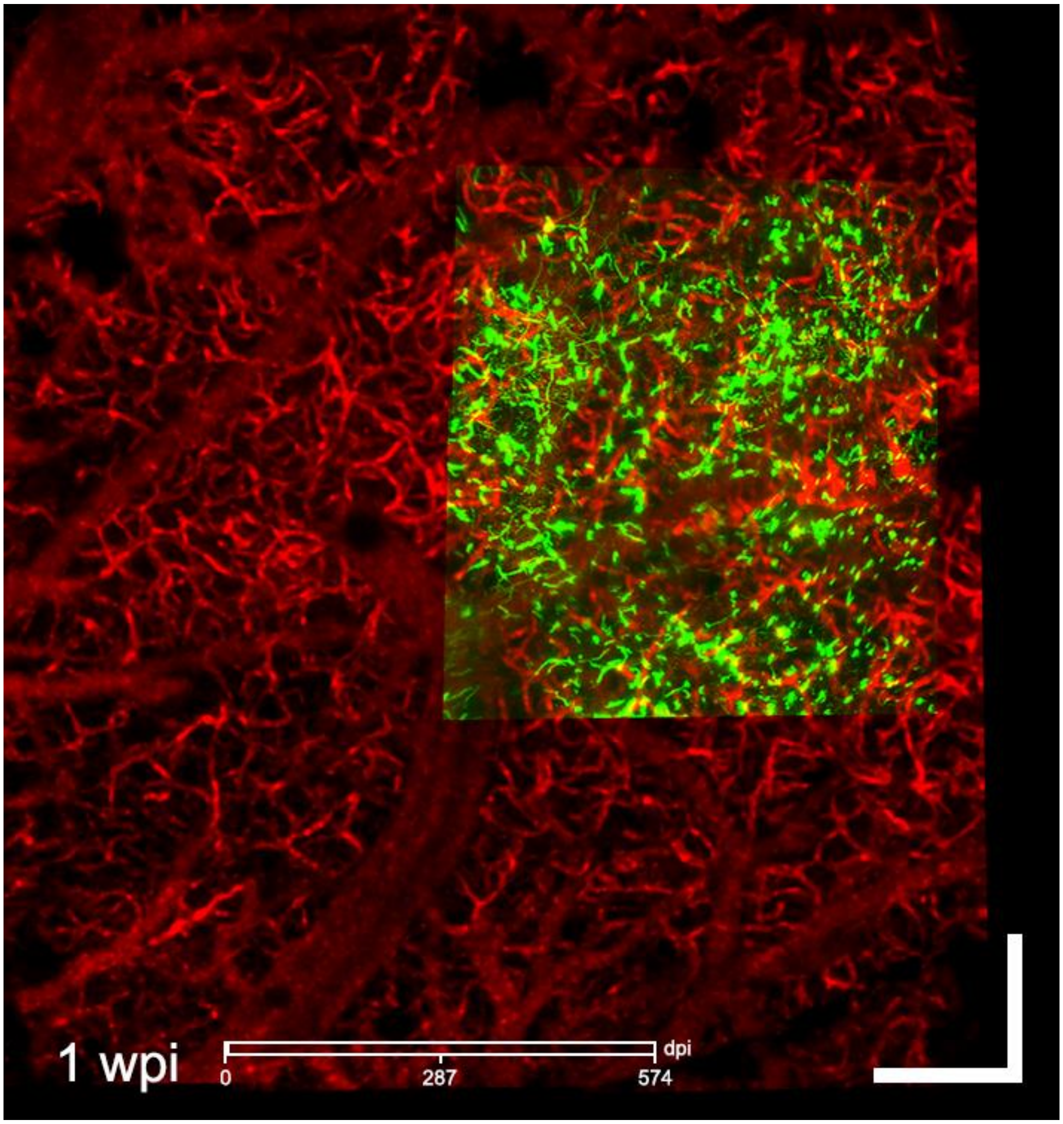

Supplemental Video 4: OCT and TPM time-lapsed videos over the experimental duration can be merge in a false color representation where red corresponds to capillaries and green corresponds to layer V neurons. Scale-bar = 200 µm.

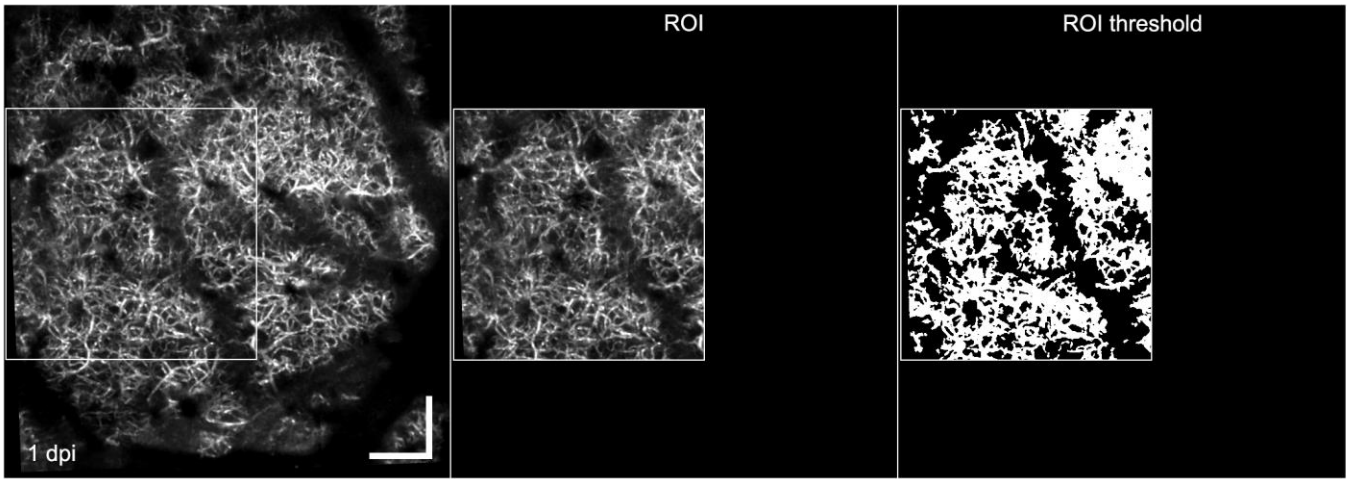

Supplemental Video 5: Time-lapsed video of deeper capillary network over one year in a window-only animal indicating ROI and thresholding for large ROI. Scale-bar = 200 µm.

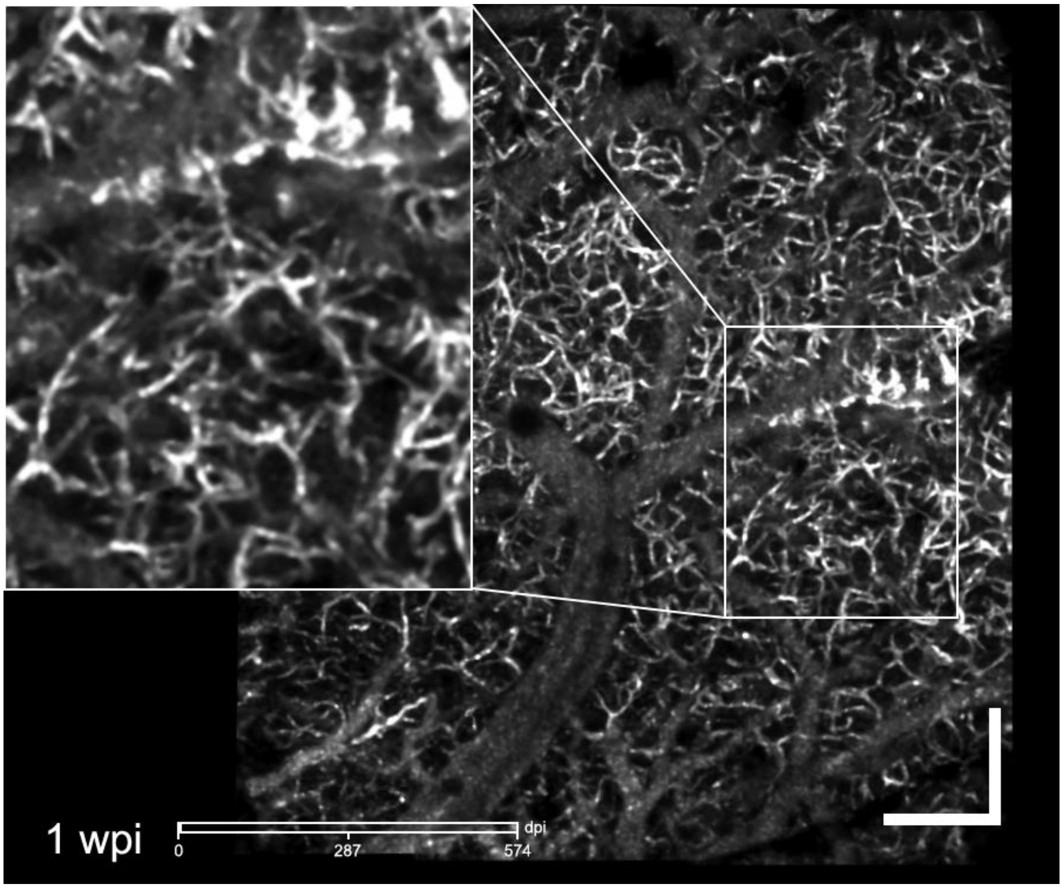

Supplemental Video 6: Time-lapsed video of deeper capillary network over ~1.5 years showing flow drop-out and other associated capillary changes around electrode starting on 29 wpi, with partial recovery on 33 wpi, and further damage at 42 wpi and later timepoints. Inset shows 2× zoomed region around the electrode for better visualization of changes. Scale-bar = 200 µm.

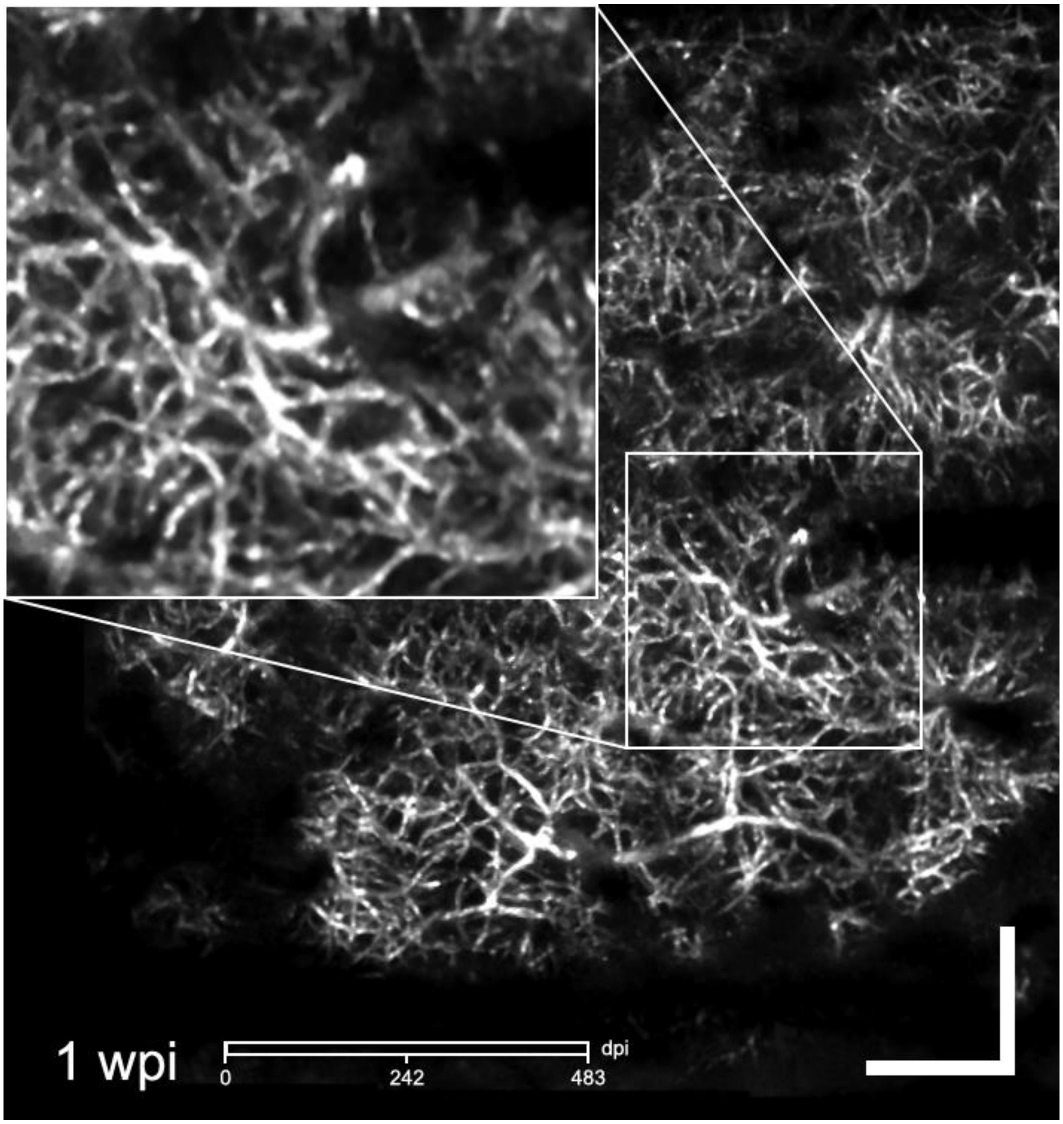

Supplemental Video 7: Time-lapsed video of deeper capillary network over ~1.3 years showing late phase capillary stretching around electrode starting on 29 wpi. The region just above the electrode shows elongated capillary segments in the direction perpendicular to the migration direction. Inset shows 2× zoomed region around the electrode for better visualization of changes. Scale-bar = 200 µm.
